# A novel CsRNF‐CsHSF24‐*CsF3′5′H2* module regulates epigallocatechin gallate biosynthesis in *Camellia sinensis*

**DOI:** 10.1111/tpj.71155

**Published:** 2026-10-09

**Authors:** Xinzhuan Yao, Hufang Chen, Haili An, Yilan Hu, Tong Li, Qi Zhao, Tengxian He, Chao Luo, Xiaofang Zeng, Litang Lu

**Affiliations:** ^1^ College of Life Sciences Guizhou University Guiyang 550025 China; ^2^ College of Tea Sciences, Institute of Plant Health & Medicine Guizhou University Guiyang 550025 China

**Keywords:** *Camellia sinensis*, epigallocatechin gallate (EGCG) biosynthesis, CsRNF, *CsHSF24*, *CsF3'5'H2*, ubiquitination, natural variation, transcriptional regulation

## Abstract

Tea is rich in tea polyphenols, vitamins, amino acids, and other components that have many benefits to health. Catechins are divided into dihydroxy catechins and trihydroxy catechins based on the hydroxylation pattern of their B‐ring. Although genes involved in catechin biosynthesis have been extensively studied and several transcription factors have been reported to regulate this process, the regulatory mechanisms controlling catechin biosynthesis remain incompletely understood. In particular, the role of post‐translational modifications of transcription factors in catechin biosynthesis is still largely unknown. In this study, we identified tea plant heat shock transcription factor (*CsHSF24*) as an important positive regulator of epigallocatechin gallate (EGCG) biosynthesis. Specifically, we found that CsHSF24 binds to the promoter of *CsF3'5'H2*, which is a key controller of EGCG biosynthesis in tea plants, as its transcriptional activator. In addition, CsRNF functions as a RING‐finger E3 ubiquitin ligase to ubiquitinate CsHSF24, leading to its degradation through the 26S proteasome pathway. Degradation of CsHSF24 reduces *CsF3'5'H2* expression, thereby inhibiting EGCG biosynthesis. Additionally, we identified a naturally occurring 14‐bp deletion in the *CsF3'5'H2* promoter of the low‐EGCG cultivar “Siqiu.” Although located ~450 bp upstream of the canonical HSE, this deletion functions as a critical structural hinge; its absence disrupts the spatial architecture required for robust transcriptional activation, thereby reducing both *CsF3'5'H2* expression and EGCG accumulation. This discovery reveals a natural structural variant that links genetic polymorphism to metabolic diversity via the *CsRNF‐CsHSF24‐CsF3'5'H2* regulatory module.

## Significance Statement

While the biosynthetic pathway of tea catechins is well established, how post‐translational modifications govern this process has remained elusive. This study uncovers a regulatory module in which the E3 ubiquitin ligase CsRNF targets the transcription factor *CsHSF24* for degradation, thereby controlling expression of the key enzyme *CsF3'5'H2* for epigallocatechin gallate biosynthesis, and identifies a natural promoter deletion that underpins metabolic variation among cultivars, offering new strategies for tea quality improvement.

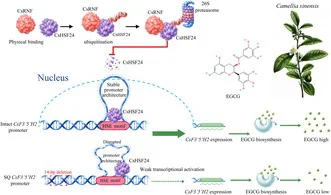

## INTRODUCTION

Tea plants (Camellia sinensis), an important cash crop widely cultivated around the world, are rich in polyphenols, theanine, vitamins, amino acids, and other components that have many benefits to health (Hayat et al., 2015). Catechins are the main functional components in tea plants, accounting for approximately 70% of total polyphenols and 12–24% of the dry weight of tea leaves; they are also key contributing factors to the bitter flavor of tea. In addition, catechins possess important pharmacological properties, including inhibition of cancer cell proliferation, induction of apoptosis, and prevention of cardiovascular and cerebrovascular diseases, owing to their strong antioxidant and free radical scavenging capacities (Kumar et al., 2015; Manikandan et al., 2012).

Catechins, derivatives of 2‐phenylbenzopyran, belong to the flavan‐3‐ols, a subclass of flavonoids, and are derived from the phenylpropanoid and flavonoid biosynthetic pathways (Wang et al., 2014; Yang et al., 2012; Zhao et al., 2020). Catechins mainly include catechin (C), epicatechin (EC), gallocatechin (GC), epigallocatechin (EGC), gallocatechin gallate (GCG), epicatechin gallate (ECG), epigallocatechin gallate (EGCG), and catechin gallate (CG) (Graham, 1992; Hong et al., 2014). Flavonoids can be divided into three subclasses including B‐ring 4′‐hydroxylation, 3′ 4′‐dihydroxylation, and 3′ 4′ 5′‐trihydroxylation compounds, based on their B‐ring hydroxylation patterns (Wang et al., 2014). In tea plant leaves, the main compounds are trihydroxy catechins (EGC, EGCG, GC) and dihydroxy catechins (C, EC, ECG), with almost no tetrahydroxy catechins; the content of trihydroxy catechins is significantly higher than that of dihydroxy catechins, and EGCG is the most abundant compound (Guo et al., 2019; Punyasiri et al., 2004; Wang et al., 2014). In plants, the phenylpropanoid and flavonoid biosynthetic pathways have been widely studied, and most of the enzymes and genes involved have been identified (Liu et al., 2021; Zhao et al., 2020). Flavonoid 3′,5′‐hydroxylase (F*3′5′*H), a member of cytochrome P450 family, is an important enzyme involved in the flavonols and flavan‐3‐ols biosynthesis pathways through catalyzing the hydroxylation of the B‐ring at the 3′ or 5′ position of flavonoids (Olsen et al., 2010; Seitz et al., 2006). Heterologous expression of *GbF3′5′H* from ginkgo increased flavan‐3‐ol (EC and GC) content in transgenic Populus (Wu et al., 2020). In tea plants, *F3′5′H* is a key controller of trihydroxylated flavan‐3‐ol synthesis: The deletion of 14 bp in the *F3′5′H2* allele downregulates *F3′5′H2* mRNA expression, leading to decreased trihydroxylated catechin (TRIC) content and an increased catechin index (CI) (Jin et al., 2018; Wang et al., 2014). Liu et al. (2020) observed that the low expression of *F3′5′H2* caused by the deletion of 14 bp in its promoter leads to reduced accumulation of trihydroxylated flavonoids in the leaves of two wild tea plants. Guo et al. further reported that the expression of *CsF3′5′Ha* and *CsF3′5′Hb* is positively correlated with B‐trihydroxylated catechin content; enzyme activity assays in yeast demonstrated that *CsF3′5′Ha* can utilize flavanones, flavonols, and flavanonols as substrates, whereas *CsF3′5′Hb* is restricted to flavonols and flavanonols (Guo et al., 2019).

Transcription factors (TFs) are key regulators that directly or indirectly bind to cis‐regulatory elements to control target gene transcription (Luo et al., 2022). With the development of molecular biotechnology, an increasing number of transcription factors regulating catechin biosynthesis in tea plants have been identified through multi‐omics (transcriptome, proteome, metabolome, etc.) analyses, such as *MYB*, *WRKY*, and TCP family members (Luo et al., 2022; Yu, Li, et al., 2021; Zhao et al., 2023). The heat shock transcription factor (*HSF*) is a kind of transcription factor that exists widely in both plants and animals (Scharf et al., 2012). HSFs are terminal components of the signal transduction chain that mediate the activation of genes in response to heat stress and/or other stresses, including drought, cold, and salinity (Guo et al., 2016; Hahn et al., 2011; Nover et al., 2001). Additionally, plant *HSFs* also have been reported to be involved in plant growth and development. Overexpression of *HSFB4* caused the root surface of transgenic plants to be rougher, and cells became detached compared with WT plants (Begum et al., 2013). Constitutive overexpression of *HSFA9*, a seed‐specific HSF from sunflower, in tobacco increased the contents of carotenoids, chlorophyllide, and chlorophyll and accelerated cotyledon unfolding (Prieto‐Dapena et al., 2017). In Petunia hybrida, *PhHSF19* was found to potentially function as a positive regulator of benzenoid/phenylpropanoid biosynthesis through regulating *PhPAL2* expression (Fu et al., 2022). Additionally, in tea plants, *CsHSFA1b* and *CsHSFA2* were shown to regulate CsJAZ6 expression by binding to its promoter, thereby modulating catechin accumulation (Zhang et al., 2023). Notably, *HSFs* have recently emerged as important regulators of flavonoid metabolism in diverse species: For example, *MdHSFA8a* modulates flavonoid synthesis and drought tolerance in apple (Wang et al., 2020), *MaHSF11* positively regulates flavonol biosynthesis and flavonoid B‐ring hydroxylation in banana (Naik et al., 2025), and a class B *HSF* selected during soybean domestication promotes flavonoid biosynthesis and salt tolerance (Bian et al., 2020). However, there are few studies on the role of *HSF* in regulating the biosynthesis of plant secondary metabolites, and its role in tea plant catechins biosynthesis is still unclear.

Protein post‐translational modifications (PTMs), including ubiquitination, phosphorylation, glycosylation, and methylation, can affect the activity of transcription factors and play critical roles in plant stress responses, growth, and development (Liu et al., 2017; Zhang et al., 2022). A growing body of evidence highlights the importance of ubiquitination in regulating TFs involved in secondary metabolism. For example, the E3 ubiquitin ligase GW2 ubiquitinates WG1, a glutaredoxin protein that promotes grain growth by increasing cell proliferation, and modulates its stability to control rice grain growth (Hao et al., 2021; Zhang et al., 2022). In apple, *MdMAPK3* and *MdPUB24* interact with *MdNAC72* and phosphorylate and ubiquitinate it, respectively; *MdNAC72* functions as a transcriptional repressor of the cell‐wall‐degrading gene POLYGALACTURONASE1 (MdPG1), and its degradation promotes ethylene‐induced fruit softening (Wei et al., 2023). In Arabidopsis, the E3 ligases RGLG1 and RGLG2 ubiquitinate MAPKKK18 and promote its degradation to modulate drought tolerance (Yu, Kang, et al., 2021). More directly relevant to flavonoid metabolism, reduced proteasome‐mediated degradation of the B‐class HSF HsfB2a at higher temperatures was recently shown to inhibit anthocyanin synthesis in pear (Wang et al., 2025). Despite these advances, little is known about the role of PTMs in modulating the activity of TFs involved in plant secondary metabolite biosynthesis, particularly in catechin biosynthesis in tea plants.

In this study, we focused on the role of *CsF3′5′H2* (*CsF3′5′Ha* in previous studies) in tea plant catechin biosynthesis. We found that *CsF3′5′H2* expression was positively correlated with EGCG accumulation. Furthermore, we identified CsHSF24 as a positive regulator of trihydroxy catechin biosynthesis that directly regulates *CsF3′5′H2* expression. We also demonstrated that CsRNF, a RING‐type E3 ubiquitin ligase, ubiquitinates CsHSF24 and targets it for degradation via the 26S proteasome pathway. Additionally, we discovered a naturally occurring 14‐bp deletion in the *CsF3′5′H2* promoter of the low‐EGCG cultivar “Siqiu” that attenuates promoter activity without directly disrupting the CsHSF24‐binding site, providing a direct link between natural genetic variation and metabolic diversity. Collectively, our findings reveal a novel CsRNF‐CsHSF24‐CsF3*′*5*′*H2 regulatory module that governs EGCG accumulation in tea plants and provide new insights into the molecular mechanisms underlying catechin biosynthesis. It should be noted that the final galloylation step of EGCG formation is catalyzed by serine carboxypeptidase‐like acyltransferases (SCPLs); however, because CsF3*′*5*′*H2 determines the supply of trihydroxylated B‐ring precursors and is the major quantitative determinant of EGCG content (Guo et al., 2019; Wang et al., 2014), we focused on the transcriptional and post‐translational regulation of *CsF3′5′H2*, which provides an entry point for understanding how the entire EGCG biosynthetic branch is controlled.

## RESULTS

### *CsF3′5′H* expression is correlated with epigallocatechin gallate accumulation in tea plants

To investigate the metabolic divergence in tea quality‐related metabolites, we quantified the levels of epigallocatechin gallate (EGCG)—the predominant trihydroxy catechin—in the leaves of four representative tea cultivars. As shown in Figure 1A, significant variations in EGCG content were observed among the tested genotypes. The cultivar “SQ” contained the lowest EGCG content (35.74 mg/g DW), which was significantly lower than that of the other three cultivars (*P* < 0.05). By contrast, “ZC108” exhibited the highest accumulation of EGCG (69.31 mg/g DW), followed by “WNZ” (65.43 mg/g DW) and “LJ43” (59.70 mg/g DW) (Figure 1A). This pronounced phenotypic variation suggests distinct genetic regulatory capacities underlying EGCG biosynthesis in these elite cultivars.

**Figure 1:**
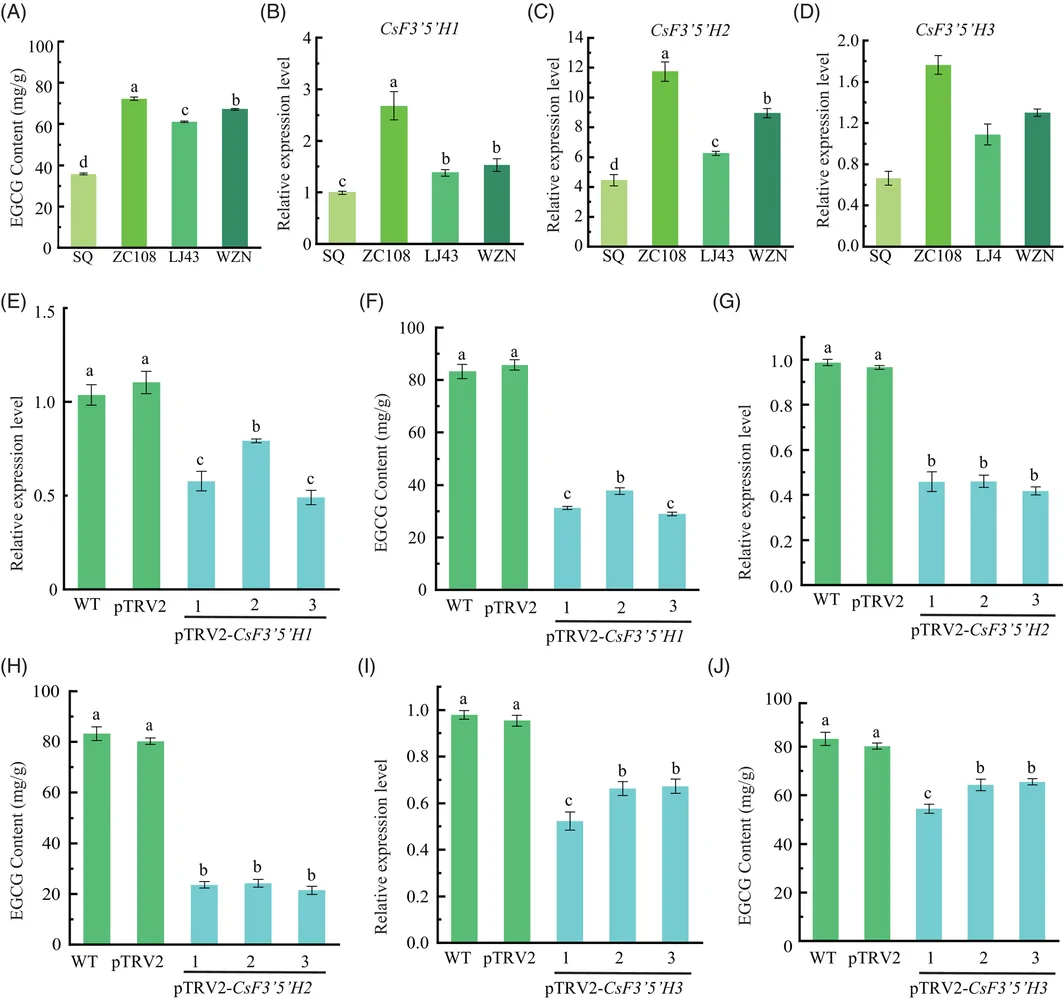
The role of *CsF3′5′H* in tea plants trihydroxy catechins biosynthesis. (A) The contents of EGCG in leaves of one wild tea plant (“Siqiu” (“SQ”)) and three tea cultivars (“Wuniuzao” (“WNZ”), “Longjing43” (“LJ43”), “Zhongcha108” (“ZC108”)); (B) expression levels of *CsF3′5′H1*, (C) *CsF3′5′H2*, and (D) *CsF3′5′H3* in leaves of the four tea cultivars (SQ, WNZ, LJ43, and ZC108). (E) Expression level of *CsF3′5′H1* and (F) EGCG content in *CsF3′5′H1*‐silenced (pTRV2‐*CsF3′5′H1*) and control (WT and pTRV2) tea plants. (G) Expression level of Cs*F3′5′H2* and (H) EGCG content in *CsF3′5′H2*‐silenced (pTRV2‐*CsF3′5′H2*) and control tea plants. (I) Expression level of *CsF3′5′H3* and (J) EGCG content in *CsF3′5′H3*‐silenced (pTRV2‐*CsF3′5′H3*) and control tea plants. WT plants are untreated plants; pTRV2 plants are plants transiently transformed with pTRV1 + pTRV2; pTRV2‐*CsF3′5′H1*, pTRV2‐*CsF3′5′H2*, and pTRV2‐*CsF3′5′H3* plants are plants transiently transformed with pTRV1 plus the corresponding pTRV2 derivative. Error bars: ±SD. Statistical significance was determined using Tukey's multiple range test (*P* < 0.05). ND, not detected.

The expression and activity of *CsF3′5′H* directly determine the composition and content of catechins in tea leaves, representing a core factor influencing tea flavor, astringency, and health‐related components. This makes it highly significant for elucidating the molecular regulatory mechanisms underlying the biosynthesis of EGCG, a key secondary metabolite in tea plants. Therefore, the expression levels of *CsF3′5′H1*, *CsF3′5′H2*, and *CsF3′5′H3* were analyzed in leaves of four tea cultivars (“WNZ,” “ZC108,” “LJ43,” and “SQ”) (Figure 1B–D). The results revealed that the expression of all three genes was highest in “ZC108,” followed by “WNZ” and “LJ43,” and lowest in “SQ.” Among them, *CsF3′5′H2* exhibited significantly higher expression than *CsF3′5′H1* and *CsF3′5′H3* across all cultivars, whereas no obvious difference was observed between *CsF3′5′H1* and *CsF3′5′H3* within the same cultivar. In addition, we examined the expression of *CsF3′5′H1*, *CsF3′5′H2*, and *CsF3′5′H3* in different tissues of the “WNZ” cultivar. All three genes showed a similar tissue‐specific expression pattern: The highest levels were detected in buds, followed by old leaves and stems, lower levels in pericarp and roots, nearly undetectable expression in flowers (Figure S1).

To investigate the individual contribution of each *CsF3′5′H* gene to catechin accumulation, we employed the TRV‐mediated VIGS system to specifically silence *CsF3′5′H1*, *CsF3′5′H2*, and *CsF3′5′H3*, respectively, in tea plants. After 40 days of infiltration, leaves from control (WT and pTRV2) and *CsF3′5′H1* silenced (pTRV2‐*CsF3′5′H1*) tea plants were harvested (Figure S4). The expression of *CsF3′5′H1* was significantly lower in *CsF3′5′H1*‐silenced (pTRV2‐*CsF3′5′H1*) plants than in wild‐type (WT) and control (pTRV2) plants (Figure 1E). Expression of *CsF3′5′H2* and *CsF3′5′H3* showed a similar pattern, with significantly lower levels in the silenced plants compared with both the wild‐type (WT) and the empty vector control (pTRV2) plants (Figure 1G,I). Consistent with the reduced gene expression, further determination of catechin contents revealed that the EGCG levels in each silenced line were significantly lower than those in the WT and control (pTRV2) plants, respectively (Figure 1F,H,J). Among the silenced lines, the strongest reduction in EGCG content was observed when *CsF3′5′H2* was silenced, where the EGCG level dropped to 23.89 mg/g DW—a decrease of 71.30% compared with the WT (83.21 mg/g DW). Silencing of *CsF3′5′H1* resulted in an EGCG content of 33.74 mg/g DW, representing a 59.45% reduction relative to the WT. By contrast, silencing *CsF3′5′H3* had the mildest effect, with EGCG content at 61.45 mg/g DW, which was only 26.15% lower than that of the WT.

These results collectively demonstrate that the *CsF3′5′H* genes play crucial regulatory roles in catechin accumulation, particularly EGCG, in tea plants.

### *CsHSF24* binds to the promoter of *CsF3′5′H*

To further elucidate the molecular regulatory mechanisms underlying catechin accumulation in tea plants, we constructed bait vectors containing the promoters of *CsF3′5′H1*, *CsF3′5′H2*, and *CsF3′5′H3*, respectively, and screened a tea plant cDNA library using the yeast one‐hybrid (Y1H) system. The results showed that a common upstream candidate regulatory factor (NCBI accession: XP_028114097.1) was identified in the promoter baits of both *CsF3′5′H1* and *CsF3′5′H2*, but not in that of *CsF3′5′H3*. This protein was annotated as a heat shock factor protein (HSF24) through NCBI analysis, and we named it CsHSF24 (Tables S2, S3). The ORF of *CsHSF24* spans 873 bp and encodes 290 amino acids with a predicted molecular weight of approximately 32.14 kDa. Sequence analysis results showed that *CsHSF24* is a member of the HSF family and has a highly conserved DNA‐binding domain with HSFs from different plant species (Figure S2). RT‐qPCR analysis revealed that the expression pattern of CsHSF24 across the four tea cultivars (“WZN,” “LJ43,” “ZC108,” and “SQ”) was similar to that of *CsF3'5'H*. Specifically, *CsHSF24* expression was significantly higher in “ZC108” than in the other three cultivars, while its relative expression was lowest in “SQ” (Figure 2A).

**Figure 2:**
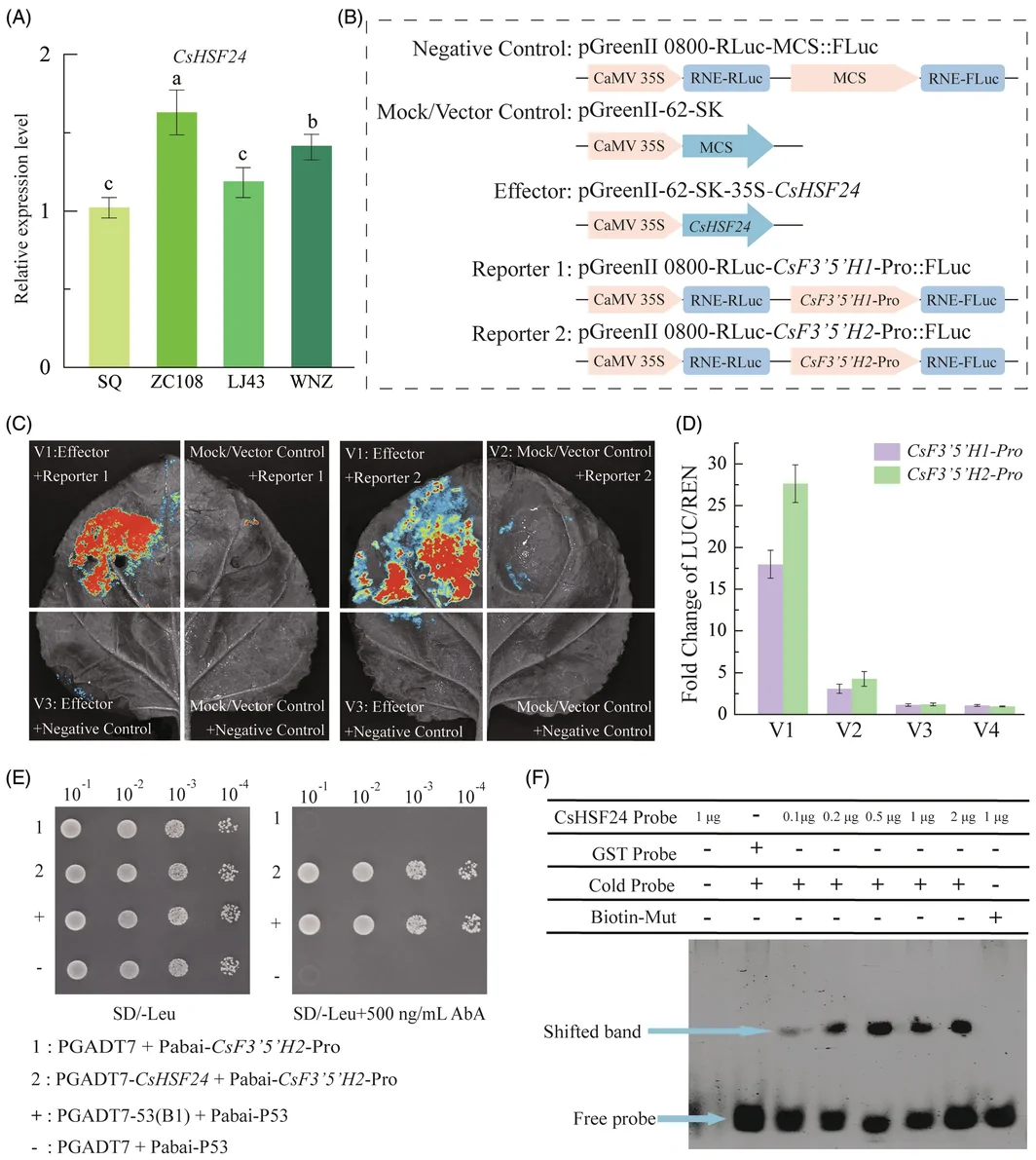
Subcellular localization of CsHSF24 and its binding to the promoter of *CsF3′5′H2*. (A) The expression level of *CsHSF24* in leaves of one wild tea plant (“Siqiu” (“SQ”)) and three tea cultivars (“Wuniuzao” (“WNZ”), “Longjing43” (“LJ43”), “Zhongcha108” (“ZC108”)); (B) schematic diagram of the dual‐luciferase reporter vector. (C) Dual‐luciferase reporter assays showing that CsHSF24 promotes the expression of *CsF3′5′H1* and *CsF3′5′H2*. (D) Promoter activities of *CsF3′5′H1* and *CsF3′5′H2* are expressed as the ratio of Firefly luciferase (LUC) to Renilla luciferase (REN) activity. (E) Y1H assay showing that CsHSF24 directly binds to the promoter of *CsF3′5′H2*. Yeast transformed with pGADT7‐CsHSF24 grew on SD/‐Leu medium containing 500 ng/mL AbA, whereas the negative control (empty pGADT7 + CsF*3′5′*H2‐Pro) did not. (F) EMSA showing that CsHSF24 directly binds the HSE motif in the *CsF3′5′H2* promoter. The unlabeled wild‐type probe was incubated alone (lane 1), with 1 μg (100×) GST as a negative control (lane 2), or with increasing amounts of GST‐CsHSF24 (5×, 10×, 50×, 100×, and 200× (1× = 0.05 μg); lanes 3–7). In lane 8, an unlabeled mutant probe carrying six base substitutions in the HSE core was incubated with GST‐CsHSF24 (1 μg, 100×). DNA was visualized by GelRed staining after electrophoresis. A shifted band appeared in a dose‐dependent manner with increasing GST‐CsHSF24 and saturated at ≥ 0.5 μg, whereas no shifted band was detected with GST alone or with the mutant probe, indicating that CsHSF24 binds sequence‐specifically to the HSE motif of the *CsF3′5′H2* promoter *in vitro*.

In addition, a dual‐luciferase reporter assay showed that a strong luminescence signal was observed during the coexpression of *35S‐Pro:CsHSF24* with *CsF3'5'H1‐Pro:LUC* and *CsF3'5'H2‐Pro:LUC*, whereas the coexpression of *35S‐Pro* with *0800‐Pro:LUC*, *35S‐Pro:CsHSF24* with *0800‐Pro:LUC*, and *35S‐Pro* with *CsF3'5'H1‐Pro:LUC* and *CsF3'5'H2‐Pro:LUC* exhibited weak or no visible luminescence signals (Figure 2B–D). Using Renilla luciferase (REN) as an internal reference, the relative transactivation activity was quantified. The results showed that the activity of 35S:CsHSF24 with CsF3*'*5*'*H2‐Pro:LUC was the highest, while its activity with CsF3*'*5*'*H1‐Pro:LUC was significantly lower (Figure 2D). These findings demonstrate that CsHSF24 can directly bind to the promoters of both CsF3*'*5*'*H1 and CsF3*'*5*'*H2. Given this marked difference, and the fact that *CsF3'5'H2* has been reported as the major contributor to trihydroxy catechin accumulation (Guo et al., 2019), we selected *CsF3'5'H2* as the primary focus for subsequent functional analyses.

We further confirmed the binding of *CsHSF24* to the *CsF3'5'H2* promoter using a Y1H assay. Yeast cells co‐transformed with pGADT7‐CsHSF24 and the promoter‐reporter construct (pAbAi‐*CsF3'5'H2*‐Pro) grew on SD/−Leu medium supplemented with 500 ng/mL AbA, whereas the negative control did not, confirming that *CsHSF24* directly binds to the *CsF3'5'H2* promoter (Figure 2E). Electrophoretic mobility shift assay (EMSA) results further validated the direct binding of CsHSF24 to the *CsF3'5'H2* promoter. Because His‐tagged CsHSF24 bound probes non‐specifically in preliminary assays (Figure S3), CsHSF24 was purified as a GST fusion protein and an unlabeled (cold) probe system was adopted for EMSA. Four probes (32–34 bp) covering different regions of the CsF3*'*5*'*H2 promoter were designed: probe 1 (−1641 to −1608 bp), probe 2 (−1699 to −1666 bp), probe 4 (−418 to −387 bp), and probe 3 (−270 to −239 bp) (Table S1), the latter containing the HSE motif HSE2 (5*'*‐GAAAAGTTCT‐3*'*, a variant of the nGAAnnTTCnn consensus) at −263 to −254 bp; probe 4 was identical to probe 3 except for six base substitutions in the HSE core (5*'*‐GAAAAGTTCT‐3*'* mutated to 5*'*‐TCCAAGAAAT‐3′) and served as the mutant‐competitor control (Figure 2F). EMSAs were performed to test whether CsHSF24 directly binds the HSE motif in the *CsF3′5′H2* promoter. No shifted band was detected when the wild‐type probe was loaded alone or incubated with 1 μg GST, indicating that neither the probe nor the GST tag produces nonspecific migration (Figure 2F). When the probe was incubated with increasing amounts of GST‐CsHSF24 (0.05 (5×), 0.1 (10×), 0.5 (50×), 1 (100×), and 2 μg (200×)), a shifted band appeared in a dose‐dependent manner and saturated at ≥ 0.5 μg, indicating direct binding of CsHSF24 to the promoter fragment. The shifted band was abolished when six base substitutions were introduced into the HSE core of the probe, even in the presence of GST‐CsHSF24 (1 μg (100×)), demonstrating that the interaction depends on the intact HSE motif. Together, these results indicate that CsHSF24 binds sequence‐specifically to the HSE element of the *CsF3′5′H2* promoter *in vitro*.

### *CsHSF24* acts as a positive regulator to regulate EGCG biosynthesis in tea plants

Subcellular localization analysis showed that the fluorescent signal of the control vector (35S:GFP) was distributed throughout the cell, whereas the 35S:*CsHSF24*‐GFP signal was predominantly detected in the nucleus, consistent with its predicted function as a transcription factor (Figure 3A).

**Figure 3:**
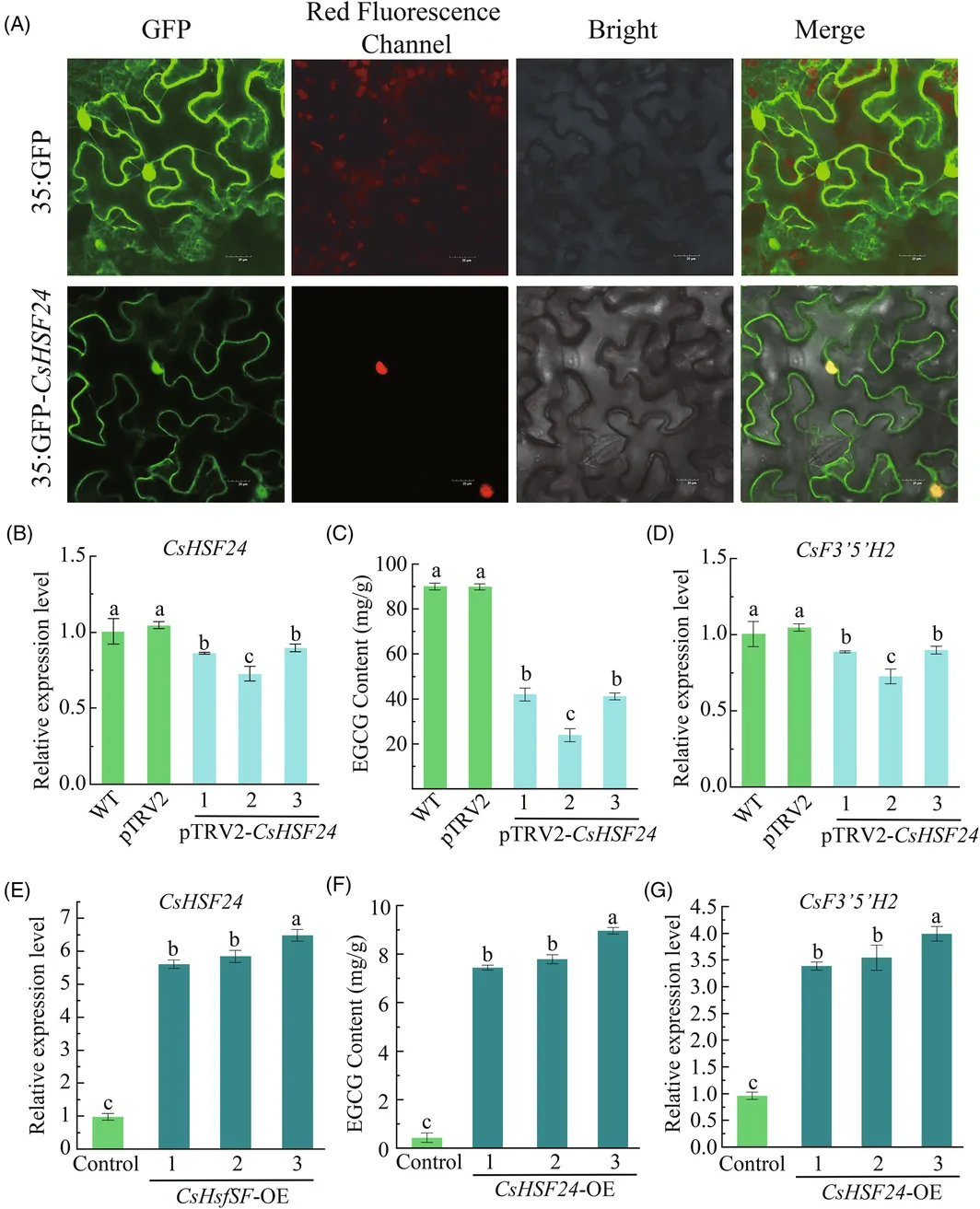
Silencing *CsHSF24* in tea plants via VIGS decreases EGCG content, whereas overexpressing it in tea callus increases EGCG content. (A) Subcellular localization of CsHSF24 in tobacco mesophyll cells. Scale bars = 20 μm. (B) The expression level of *CsHSF24* in *CsHSF24*‐silencing (pTRV2‐*CsHSF24*) and control tea plants (WT and pTRV2). (C) EGCG content in *CsHSF24*‐silencing (pTRV2‐*CsHSF24*) and control tea plants (WT and pTRV2). (D) Expression level of *CsF3′5′H2* in *CsHSF24*‐silencing (pTRV2‐*CsHSF24*) and control tea plants (WT and pTRV2). (E) *CsHSF24* expression level in *CsHSF24*‐overexpression (*CsHSF24*‐OE) and control callus. (F) EGCG content and (G) *CsF3′5′H2* expression level in *CsHSF24*‐overexpression (*CsHSF24*‐OE) and control callus. WT plant is referring to untreated plants, pTRV plant is referring to plant transient expression with pTRV1 + pTRV2, and pTRV2‐*CsHSF24* plant is referring to plant transient expression with pTRV1 + pTRV2‐*CsHSF24*. Control refers to untreated tea plant callus, while CsHSF24‐OE refers to CsHSF24‐overexpressing tea plant callus generated via *A. tumefaciens*‐mediated transformation. Error bars: ±SD. Statistical significance was determined using Tukey's multiple range test (*P* < 0.05).

Further, a TRV‐mediated VIGS system was used to silence *CsHSF24* expression in tea plants (Figure S4). Compared with wild‐type (WT) and control (pTRV2) plants, the expression level of *CsHSF24* in silenced plants decreased by 28.57% (Figure 3B), and EGCG content was significantly decreased by 53–73% (Figure 3C). Simultaneously, the expression level of *CsF3'5'H2* was also decreased to varying degrees in the *CsHSF24*‐silenced plants (Figure 3D). We next generated *CsHSF24* overexpression tea calli using *A. tumefaciens*‐mediated transformation (Figure S5). *CsHSF24* transcript levels were significantly higher in the *CsHSF24‐*overexpression lines (OE‐1, OE‐2, OE‐3) than in the control (Figure 3E). Determination of catechin contents in the different callus lines showed that EGCG content was obviously increased to varying degrees (Figure 3F). Meanwhile, *CsF3'5'H2* expression was markedly higher in the *CsHSF24*‐overexpression lines than in the control (Figure 3G), indicating that *CsHSF24* acts as a positive regulator of *CsF3'5'H2*. Based on these findings, we conclude that *CsHSF24* promotes EGCG biosynthesis in tea plants by regulating *CsF3'5'H2* transcription. To determine whether *CsHSF24* also influences other structural genes of the catechin pathway, we examined the expression of *CsF3'5'H1*, *CsF3'5'H3*, *CsDFR*, *CsANR*, *CsLAR*, and *CsANS* in the CsHSF24‐VIGS and CsHSF24‐OE samples (Figures S6a–f and S7a–f, respectively). Silencing CsHSF24 reduced the expression of *CsDFR*, *CsANR*, *CsLAR*, *CsF3'5'H1*, and *CsF3'5'H3* by 18.4%, 14.2%, 25.6%, 16.4%, 14.7%, and 16.2% relative to the pTRV2 control, respectively, whereas *CsHSF24* overexpression upregulated *CsDFR*, *CsANR*, *CsANS, CsF3'5'H1*, and *CsF3'5'H3* by 41.0%, 15.8%, 22.1%, 162.4%, and 128.3% relative to WT, respectively (CsLAR, +4.0%; Figure S7a–f). These coordinated changes in both gain‐ and loss‐of‐function samples indicate that *CsHSF24* exerts broader control over the catechin biosynthetic pathway beyond *CsF3'5'H2*. Consistent with these transcriptional changes, the contents of the major catechin fractions were also altered. In the *CsHSF24*‐silenced plants, the contents of GC, EGC, C, EC, and GCG decreased by 5.3%, 11.8%, 1.8%, 10.4%, and 33.8% relative to the pTRV2 control, respectively, whereas ECG increased by 11.0% (Figure S6g–l). Conversely, CsHSF24 overexpression increased the contents of all six catechin fractions to varying degrees, with the most pronounced increases in ECG (by 83.2%), GCG (by 47.0%), and GC (by 20.5%), followed by EC (by 18.9%), EGC (by 19.6%), and C (by 3.3%) (Figure S7g–l), indicating that stabilization of CsHSF24 promotes catechin accumulation in general.

### *CsF3'5'H2* expression and its promoter variation are correlated with epigallocatechin gallate accumulation in tea plants

Our experiments demonstrated that *CsHSF24* positively regulates EGCG biosynthesis in tea plants through modulating *CsF3′5′H2* expression. Nevertheless, the genetic basis underlying the differential expression levels of *CsF3′5′H2* among the four tea cultivars “WNZ,” “ZC108,” “LJ43,” and “SQ” remained unclear. Jin et al. identified a 14 bp deletion in the upstream regulatory region of a *CsF3′5′H2* allele in high‐EGCG wild tea resources from Guizhou, China, which was associated with reduced gene expression and consequently lower EGCG accumulation (Jin et al., 2018). Based on this finding, we hypothesized that similar structural variations in the promoter region might explain the differential *CsF3′5′H2* expression and the low‐EGCG phenotype observed in certain cultivars.

To investigate the cause of the low *CsF3′5′H2* expression in “SQ,” we cloned and sequenced both the coding and promoter regions (approximately 2.0 kb upstream of the transcription start site) from all five cultivars. Sequence alignment revealed that the coding sequences of *CsF3′5′H2* were identical across cultivars (Figure S8). By contrast, promoter analysis identified a 14‐bp deletion within a putative cis‐regulatory element. This deletion was exclusively present in the ‘SQ’ promoter and was absent in the high expression cultivars “WNZ,” “LJ43,” and “ZC108” (Figure 4A). Notably, although the 14‐bp deletion (ATTACTACACCACC) in the *CsF3′5′H2* promoter is located approximately 450 bp upstream of the typical HSF‐binding site and lacks a recognizable HSE motif (5’‐AGAAnnTTCT‐3′), it likely functions as a critical “structural hinge” mediating DNA flexibility. We propose that transcriptional activation relies not merely on the linear presence of cis‐elements, but rather on the precise three‐dimensional reconstruction of chromatin architecture. While not directly bound by *CsHSF24*, this 14‐bp sequence appears indispensable for maintaining the proper spatial conformation of *CsF3′5′H2* required for effective transcriptional activation. These findings are consistent with recent analyses showing that natural promoter variation of *F3′5′H* correlates with catechin diversification across Thea species of the genus *Camellia* (Zhang et al., 2025). In addition, we cloned and compared the *CsF3′5′H1* promoter sequences of the five cultivars; unlike *CsF3′5′H2*, no structural variation corresponding to the 14‐bp deletion was detected among them (Figure S8), whereas alignment of the *CsF3′5′H2* promoters confirmed that the 14‐bp segment is present in “WNZ,” “LJ43,” “ZC108,” and “FDDB” but absent from “SQ” (Figure S9), which is consistent with the comparable *CsF3′5′H1* expression levels across cultivars (Figure 1B) and supports *CsF3′5′H2* as the functionally relevant target of this natural variation.

**Figure 4:**
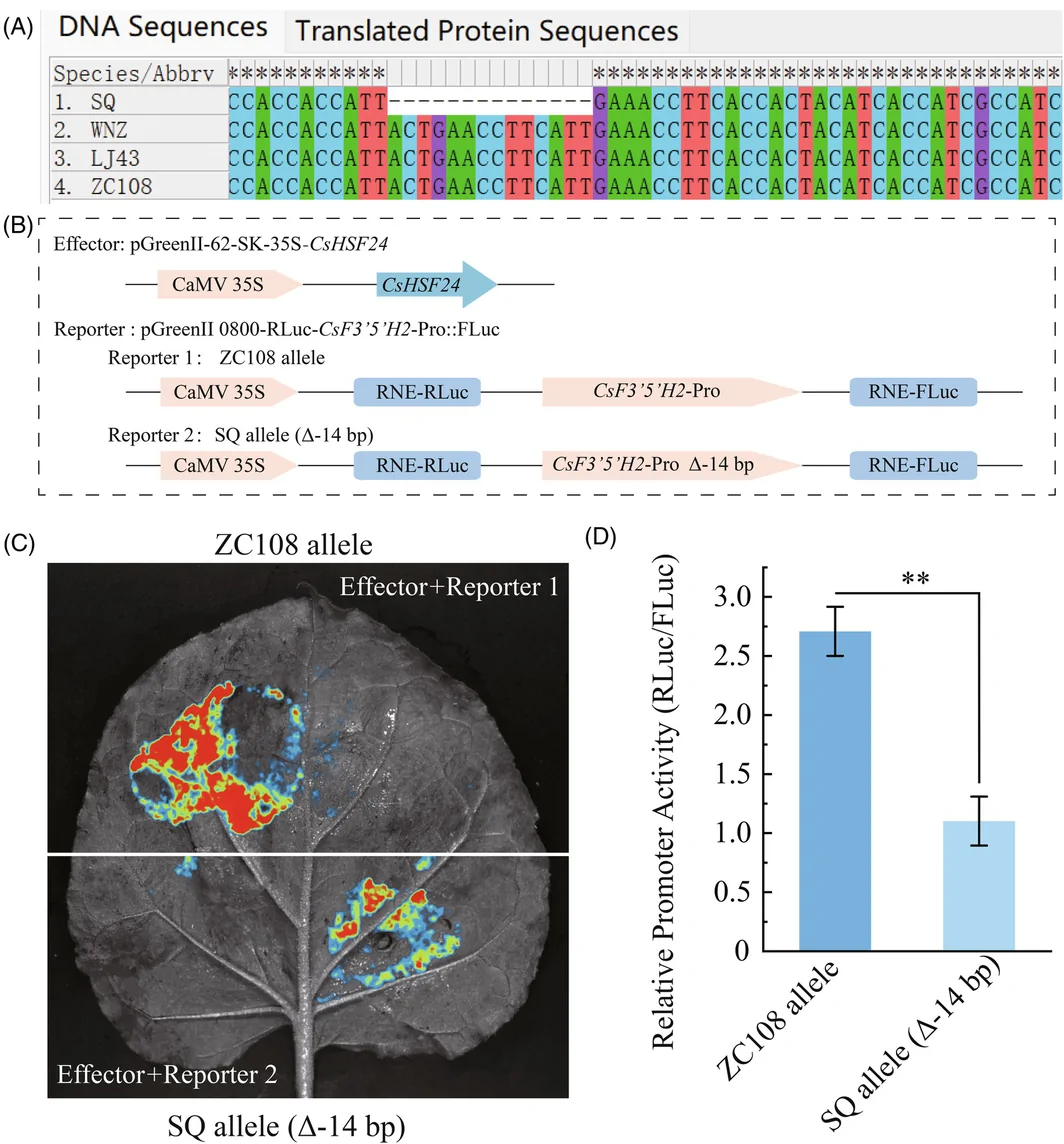
A 14 bp deletion in the *CsF3′5′H2* promoter attenuates its transcriptional activity. (A) Sequence alignment of the *CsF3′5′H2* promoter region among the four teas (“Siqiu” (“SQ”), “Wuniuzao” (“WNZ”), “Longjing43” (“LJ43”), and “Zhongcha108” (“ZC108”)). A specific Δ‐14 bp deletion was identified in the promoter region of the low expression cultivar “SQ.” (B) Schematic of the Dual‐Luciferase Reporter Assay construct. (C) Dual‐luciferase reporter assays demonstrating that the promoter activity of the CsF*3′5′*H2 allele from “SQ” was significantly lower than that of the allele from “ZC108.” (D) Promoter activity expressed as the ratio of firefly luciferase (FLuc) to Renilla luciferase (RLuc) activity. ZC108 allele represents the *F3′5′H2* promoter from the “ZC108” cultivar, and SQ allele (Δ‐14 bp) represents the *CsF3′5′H2* promoter allele harboring the 14 bp deletion from the “SQ” cultivar. Error bars: ±SD. Statistical significance was determined using Tukey's multiple range test (**P < 0.01).

To functionally validate the effect of this deletion, dual‐luciferase reporter assays were conducted. The full‐length *CsF3′5′H2* promoter sequences from “ZC108” (containing the 14‐bp segment) and “SQ” (lacking the 14‐bp segment) were cloned and fused to the firefly luciferase (Luc) reporter gene. The promoter activity of the “SQ” allele was significantly lower than that of the “ZC108” allele (Figure 4B–D), confirming that the Δ14‐bp deletion indeed reduces the transcriptional activity of the *CsF3′5′H2* promoter. This natural variation thus provides a molecular explanation for the constitutively low expression of *CsF3′5′H2* and the correspondingly low‐EGCG accumulation in the “SQ” cultivar.

### Overexpression of *CsHSF24* promotes flavonoids accumulation in heterologous tobacco system

To further validate the conserved regulatory function of CsHSF24 in flavonoids metabolism, we heterologously expressed *CsHSF24* in tobacco (*Nicotiana tabacum*) (Figure S10). Compared with the wild‐type (WT), the contents of five flavonoids rutin, quercetin, dihydroquercetin, myricetin, and dihydromyricetin were significantly increased in the transgenic lines. Among them, the increase in dihydromyricetin was the most pronounced (10.31‐ to 13.04‐fold higher than WT), followed by quercetin, dihydroquercetin, myricetin, and rutin (Figure 5). Consistent with the metabolic changes, RT‐qPCR analysis showed that the expression levels of key flavonoids biosynthetic genes, including *NtF3H*, *NtCHI*, *NtFLS*, and *NtANS*, were significantly higher in the transgenic plants (TP‐1, TP‐2, TP‐3) than in the WT plants (Figure S11). Although tobacco does not naturally synthesize EGCG due to the absence of a functional galloyltransferase, the marked increase in flavonoid intermediates upstream of the catechin pathway strongly supports the conserved role of *CsHSF24* as a positive regulator of flavonoid metabolism across plant species. These heterologous expression data corroborate the role of *CsHSF24* as a positive regulator of the flavonoids pathway. Furthermore, the expression of endogenous tobacco flavonoid biosynthetic genes was analyzed in the transgenic lines: NtDFR, NtLAR, NtANR, NtANS, NtFLS, NtF3H, and NtCHI were all significantly upregulated in TP‐1, TP‐2, and TP‐3 compared with WT (Figure S12), supporting that *CsHSF24* enhances general flavonoid biosynthesis in the heterologous host.

**Figure 5:**
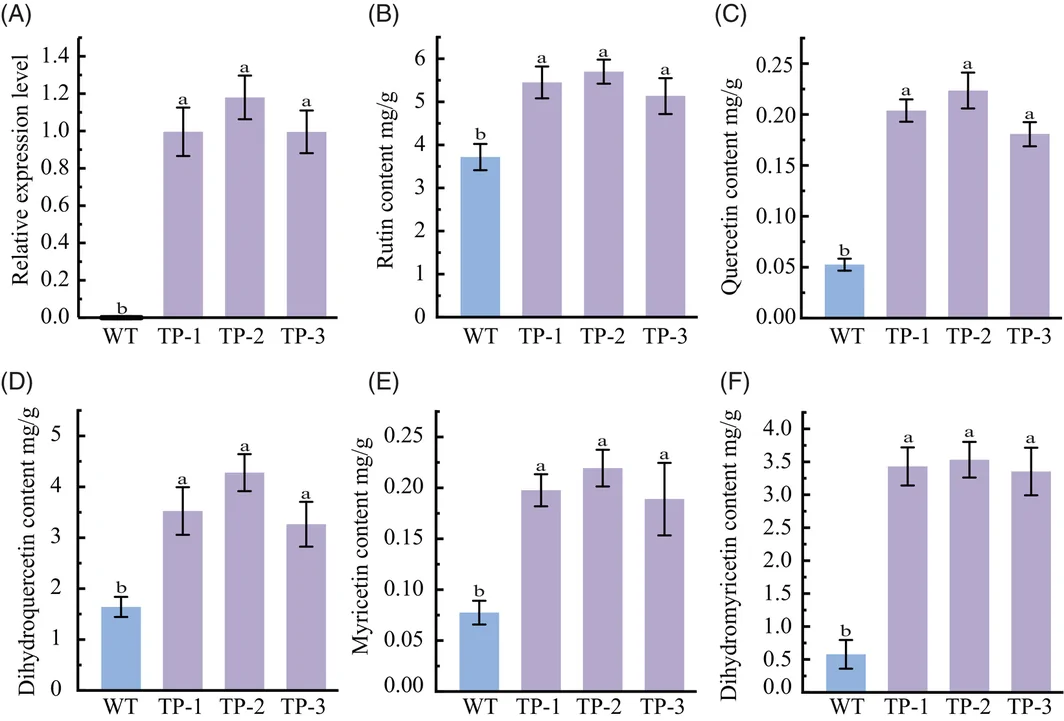
The effect of CsHSF24 overexpression on flavonoids accumulation in tobacco. (A) Relative expression level of CsHSF24 in wild‐type (WT) and transgenic tobacco lines (TP‐1, TP‐2, TP‐3). (B–F) Content of rutin (B), quercetin (C), myricetin (D), dihydromyricetin (E), and dihydroquercetin (F) in WT and transgenic tobacco lines (TP‐1, TP‐2, TP‐3). Error bars represent ± SD. Statistical significance was determined using Tukey's multiple range test (*P* < 0.05), with different lowercase letters indicating significant differences.

### CsRNF interacts with CsHSF24

To elucidate the in‐depth mechanism underlying CsHSF24 regulation of catechins biosynthesis in tea plants, we performed a yeast two‐hybrid (Y2H) screen of a cDNA library to identify putative interacting partners of CsHSF24. A total of 34 positive clones were identified (Figure S13, Table S4); sequencing and sequence comparison showed that they all encoded the same E3 ubiquitin ligase protein. Therefore, we selected this E3 ubiquitin ligase for further study. The ORF of this E3 ubiquitin ligase was 1011 bp, encoding 336 amino acids. Sequence analysis results showed that the E3 ubiquitin ligase we screened had a conserved domain of the really interesting new gene (RING) finger type E3 ubiquitin ligase and had high similarity to RNF proteins from other species; we therefore named it CsRNF (Figure S14).

To investigate the potential interaction between CsRNF and CsHSF24, we first performed a bimolecular fluorescence complementation (BiFC) assay in *N. benthamiana* leaves. When YFPN‐*CsHSF24* was co‐expressed with YFPC‐*CsRNF*, strong YFP fluorescence was observed exclusively in the nucleus (Figure 6A). By contrast, no YFP signal was detected in the negative control combinations (YFPN‐*CsHSF24* + YFPC‐EV, YFPN‐EV + YFPC‐*CsRNF*, and YFPN‐EV + YFPC‐EV), indicating that CsRNF specifically interacts with CsHSF24 in the plant nucleus. To further confirm this interaction, we conducted a yeast two‐hybrid assay. The full‐length CsRNF was fused to the GAL4 DNA‐binding domain (BD‐CsRNF), and CsHSF24 was fused to the GAL4 activation domain (AD‐CsHSF24). Yeast cells co‐transformed with BD‐CsRNF and AD‐CsHSF24 grew well on SD/‐Ade/‐His/‐Leu/‐Trp selective medium, whereas the negative control combinations did not (Figure 6B). Serial dilution assays confirmed the specificity and strength of the interaction.

**Figure 6:**
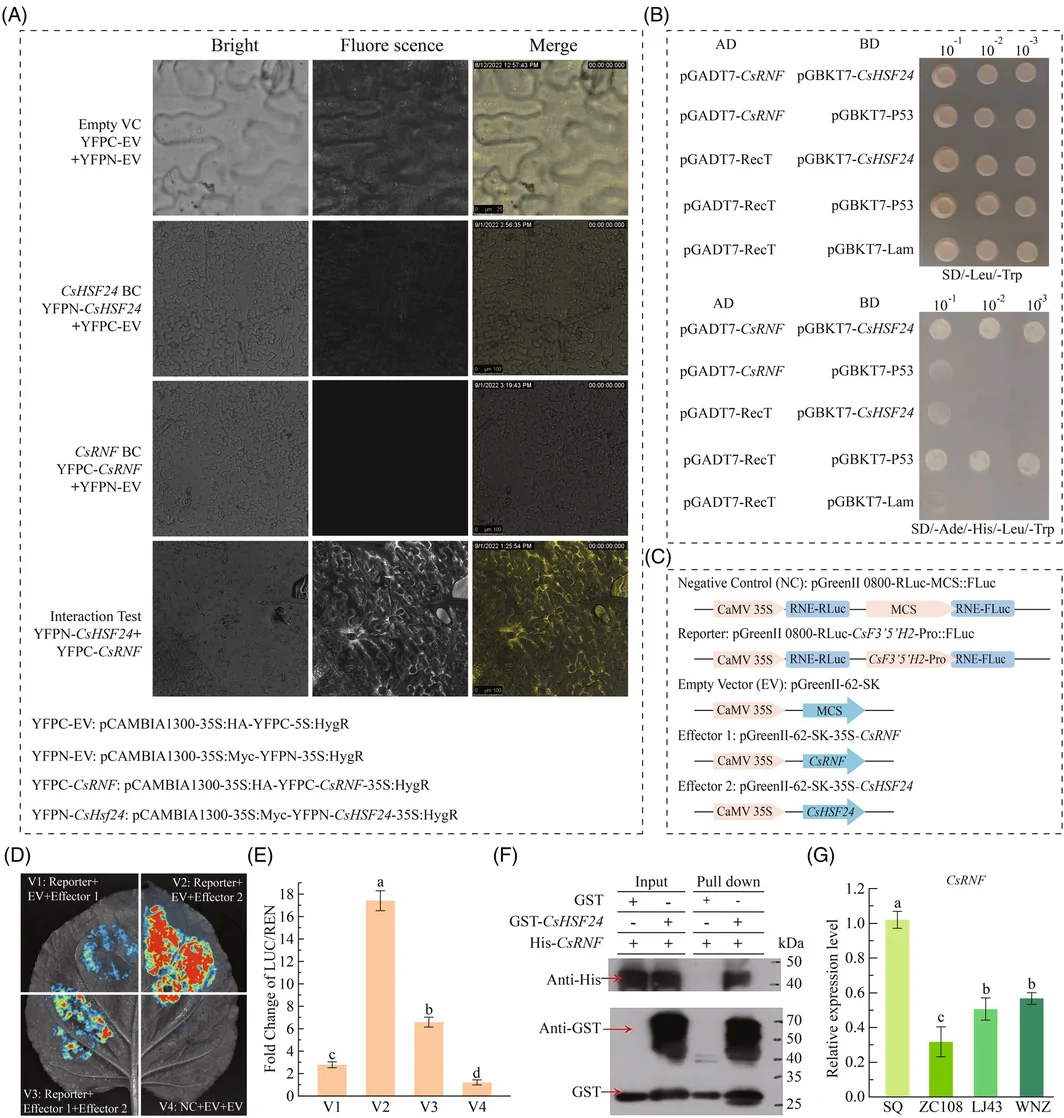
CsRNF interacts with CsHSF24 in vitro and in vivo. (A) BiFC assay demonstrating the interaction between CsHSF24 and CsRNF in *N. benthamiana* leaf cells. YFP fluorescence indicates successful interaction. Scale bar = 50 μm. EV = empty vector control; CsHSF24‐BC/CsRNF‐BC = background controls for CsHSF24 and CsRNF, respectively. Interaction test: Coexpression of YFPN‐CsHSF24 and YFPC‐CsRNF. (B) Yeast two‐hybrid assay confirming the interaction between CsRNF and CsHSF24. Serial dilutions (10^−1^ to 10^−5^) of yeast cultures were spotted onto selective media. (C) Schematic diagrams of the reporter and effector constructs used in dual‐luciferase assays. (D, E) Dual‐luciferase assay showing that CsRNF represses CsHSF24‐mediated activation of the *CsF3′5′H2* promoter. (F) A pull‐down assay of the interaction between CsRNF and CsHSF24. Recombinant GST‐CsHSF24 was incubated with immobilized His‐CsRNF and detected for immunoblot analysis using an anti‐His antibody (upper panel) or an anti‐GST antibody (bottom panel). The arrow represents the target protein. (G) The expression level of *CsRNF* in leaves of one wild tea plant (“Siqiu” (“SQ”)) and three tea cultivars (“Wuniuzao” (“WNZ”), “Longjing43” (“LJ43”), “Zhongcha108” (“ZC108”)). Error bars: ±SD. Statistical significance was determined using Tukey's multiple range test (*P* < 0.05).

Because HSF transcription factors are known to activate target gene expression and CsRNF interacts with CsHSF24, we performed dual‐luciferase reporter assays to examine whether CsHSF24 activates the *CsF3′5′H2* promoter and whether CsRNF modulates this activity. The *CsF3′5′H2* promoter was fused to the firefly luciferase (FLuc) reporter gene, and the effector constructs contained CsHSF24 and CsRNF driven by the CaMV 35S promoter (Figure 6C). When the reporter (NC: pGreenII 0800‐RLuc‐*CsF3′5′H2*‐Pro::FLuc) was co‐expressed with 35S‐*CsHSF24* (pGreenII‐62‐SK‐35S‐CsHSF24) alone (V2), the relative LUC/REN activity was significantly increased by approximately 14.35‐fold compared with the control V1: reporter + 35S‐CsRNF (pGreenII‐62‐SK‐35S‐*CsRNF*) + EV (pGreenII‐62‐SK) (Figure 6D,E). However, when 35S‐CsRNF was co‐expressed with 35S‐*CsHSF24* (V3), the LUC/REN activity was markedly reduced to approximately 5.43‐fold, indicating that CsRNF negatively regulates the transcriptional activation activity of *CsHSF24*. The negative control (V4: NC + EV) showed only background LUC activity.

A pull‐down assay verified the CsHSF24 and CsRNF could interact with each other. In the pull‐down assay, the signal of GST protein and His fusion protein (His‐CsRNF) was used as negative control. When GST‐CsHSF24 was used as bait, only His‐CsRNF protein was immunodetected using anti‐His, whereas GST empty protein had no His‐CsRNF protein signal (Figure 6F). These above results support that CsHSF24 and CsRNF interact with each other in *vitro* and in *vivo*. We further examined the expression levels of CsRNF in four tea plant germplasms with different genetic backgrounds (SQ, ZC108, LJ43, and WNZ). Quantitative real‐time PCR analysis revealed that CsRNF expression was highest in SQ, significantly lower in ZC108, and moderate in LJ43 and WNZ (Figure 6G). These results suggest that CsRNF expression is differentially regulated among tea plant germplasms, which may contribute to the natural variation in flavonoid biosynthesis through modulation of CsHSF24 stability.

### Ubiquitination of CsHSF24 by CsRNF targets it for 26S proteasome‐mediated degradation, suppressing EGCG biosynthesis in tea plants

We next verified whether CsRNF can ubiquitinate the CsHSF24 protein using an in vitro ubiquitination assay. After incubating the recombinant CsRNF‐His and CsHSF24‐GST in a reaction mixture containing ATP, Ub, E1, and E2, the reaction mixture was immunoblotted using anti‐Ub antibody. In the presence of CsRNF‐His, CsHSF24‐GST, ATP, Ub, E1, and E2, the high molecular‐mass bands corresponding to ubiquitinated CsHSF24 were detected (Figure 7A).

**Figure 7:**
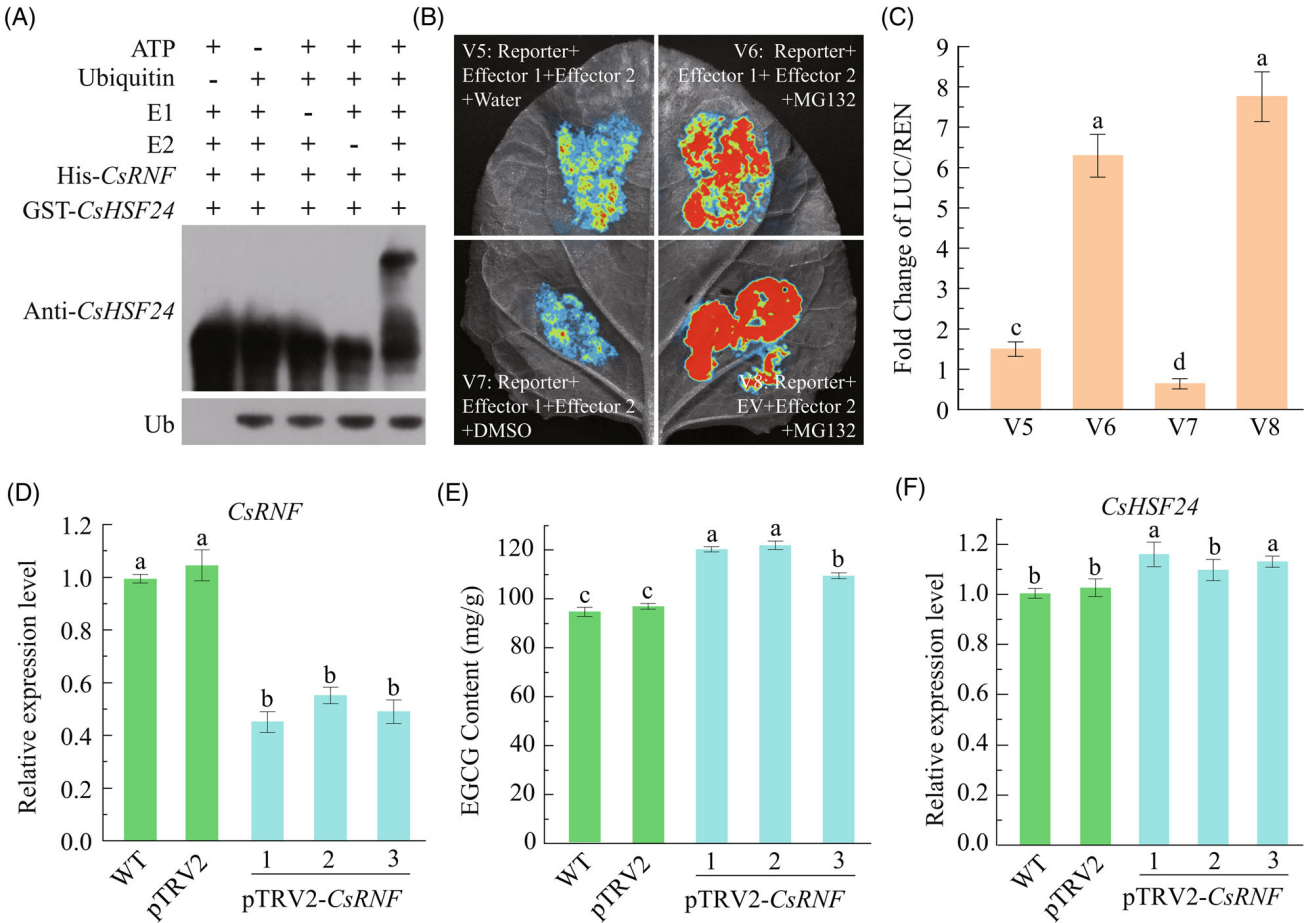
CsRNF ubiquitinates CsHSF24 and inhibits tea plants' EGCG biosynthesis. (A) CsRNF ubiquitinates CsHSF24 in *vitro*. GST‐CsHSF24 and His‐CsRNF fusion proteins were assayed for ubiquitination activity in the presence of ATP, E1, E2, and ubiquitin. Ubiquitinated proteins were detected by immunoblotting with an anti‐ubiquitin (Ub) antibody. (B) Schematic diagrams of the reporter and effector constructs used in the dual‐luciferase assay. (C) Dual‐luciferase assay with MG132 treatment demonstrating that CsRNF promotes CsHSF24 degradation via the 26S proteasome pathway. (D) Relative expression of *CsRNF* in *CsRNF*‐silencing (pTRV2‐*CsRNF*) and control tea plants (WT and pTRV2). (E) EGCG content in *CsRNF*‐silencing (pTRV2‐*CsRNF*) and control tea plants (WT and pTRV2). (F) *CsHSF24* expression level in *CsRNF*‐silencing (pTRV2‐*CsRNF*) and control tea plants (WT and pTRV2). WT plant is referring to untreated plants, pTRV plant is referring to plant transient expression with pTRV1 + pTRV2; and pTRV2‐*CsRNF* and pTRV2‐*CsHSF24* plant is referring to plant transient expression with pTRV1 + pTRV2‐*CsRNF* and pTRV1 + pTRV2‐*CsHSF24*. Error bars: ±SD. Statistical significance was determined using Tukey's multiple range test (*P* < 0.05). ND, not detected.

To determine whether CsRNF regulates CsHSF24 through the ubiquitin‐26S proteasome pathway, we repeated the dual‐luciferase assay in the presence of MG132, a specific inhibitor of the 26S proteasome. When leaves were treated with MG132 (V6), the inhibitory effect of CsRNF on CsHSF24‐mediated transcriptional activation was significantly attenuated, and LUC/REN activity was restored to a level similar to that observed when CsHSF24 was expressed alone (V8; Figure 7B,C). By contrast, treatment with water (V5) or DMSO (V7, solvent control) had no such effect. These results strongly suggest that CsRNF promotes the degradation of CsHSF24 via the 26S proteasome pathway, thereby negatively regulating its transcriptional activity.

Considering that CsRNF can ubiquitinate CsHSF24, we examined the function of CsRNF in catechin biosynthesis by silencing CsRNF in tea plants using VIGS. After 40 days of injection with *Agrobacterium* harboring pTRV2‐*CsRNF*, the leaves of *CsRNF‐silenced* (pTRV2‐*CsRNF*) and control (WT, pTRV) tea plants were harvested. RT‐qPCR analysis revealed that *CsRNF* was successfully silenced in tea plants with the expression level decreased to 45%, 55%, and 49% of the control level, respectively (Figure 7D). Determination of the catechin contents showed that EGCG content was increased by approximately 1.2‐fold compared with the control plants (Figure 7E; Figure S10). By contrast, the expression level of CsHSF24 in CsRNF‐silenced plants showed no significant change compared with WT and control (pTRV2) plants (Figure 7F), consistent with a post‐translational mode of regulation. Collectively, these findings demonstrate that CsRNF does not suppress CsHSF24 transcription but rather promotes the ubiquitination‐mediated degradation of CsHSF24 protein, thereby negatively regulating EGCG biosynthesis in tea plants.

## DISCUSSION

### *CsF3′5′H2* is the major contributor to trihydroxy catechin biosynthesis in tea plants

Tea plants are important non‐alcoholic beverage crops, and the tea produced from their buds and leaves is not only rich in flavor but also contains various substances beneficial to human health, making it popular worldwide. Among them, catechins, a polyphenol compound, is a substance that accounts for the largest proportion in fresh leaves of tea plants and dry tea, and is also a key factor determining the taste of dry tea (Yoshida et al., 1999). In tea plant leaves, the main compounds are trihydroxy catechins (EGC, EGCG, GC) and dihydroxy catechins (C, EC, ECG), with almost no tetrahydroxy catechins; the content of trihydroxy catechins is significantly higher than that of dihydroxy catechins, and EGCG is the most abundant compound (Guo et al., 2019; Punyasiri et al., 2004; Wang et al., 2014). Catechin is a type of flavonoid, with the highest total amount in tea leaves, followed by stems, and only a small amount in roots (Liu et al., 2009). In tea leaves, catechin content is highest in buds and young leaves and gradually decreases with increasing leaf maturity (Guo et al., 2019).

Until now, there have been many relevant reports on the research of tea plants catechin biosynthesis pathway, and this pathway has been relatively clear (Eungwanichayapant & Popluechai, 2009; Hong et al., 2014). F*3′5′*H is one of the key enzymes in the catechin biosynthetic pathway; it catalyzes the hydroxylation of the 3′ and 5′ positions of the flavanone B‐ring to generate the corresponding dihydroflavonols, which are important intermediate substrates for the biosynthesis of catechins and anthocyanidins (Li, Yang, et al., 2022; Wang et al., 2014). In the published tea plant genome, it was found that F*3′5′*H was encoded by two duplicate genes, *CsF3′5′Ha* (*CsF3′5′H2*) and *CsF3’5Hb* (*CsF3′5′H1*). Enzymes encoded by duplicated genes are generally functional and often exhibit redundancy or complementarity (Tian et al., 2003; Tian & DellaPenna, 2001). Research has shown that the expression levels of *CsF3′5′Ha* and *CsF3*′*5*′*Hb* were positively correlated with the content of trihydroxy catechins, and the correlation coefficient between *CsF3′5′Ha* and trihydroxy catechins content was higher (Guo et al., 2019). The low accumulation of trihydroxy flavonoid compounds in tea plants may be due to the lack of 14 bp in the *CsF3′5′H* (*CsF3′5′Ha*) promoter region, resulting in low expression levels of *CsF3′5′H* (Liu et al., 2020). Although the function of CsF*3′5′*Hb (CsF*3′5′*H1 in this study) in trihydroxy catechin biosynthesis has rarely been studied, both CsF*3′5′*Ha (CsF*3′5′*H2 in this study) and CsF*3′5′*Hb show tissue‐specific expression, with the highest levels in buds and very low levels in roots, and their expression is positively associated with the concentration of B‐trihydroxylated catechins (Guo et al., 2019; Wang et al., 2014). Our results, showing that silencing *CsF3′5′H2* caused a more pronounced reduction in EGCG than silencing *CsF3′5′H1*, are consistent with this model and reinforce the conclusion that *CsF3′5′H2* is the predominant contributor to trihydroxy catechin biosynthesis in tea leaves. It should be noted that the final galloylation of EGCG is catalyzed by serine carboxypeptidase‐like acyltransferases (SCPLs), which are also important for EGCG accumulation; nevertheless, CsF*3′5′*H2 determines the supply of the trihydroxylated B‐ring precursors and is the major quantitative determinant of EGCG content (Guo et al., 2019; Wang et al., 2014), justifying its selection as the focus of this regulatory study. Whether the SCPL genes are also subject to regulation by the CsRNF–CsHSF24 module warrants future investigation.

### *CsHSF24* directly activates *CsF3′5′H2* transcription

To dissect the upstream regulatory mechanisms of EGCG biosynthesis, we used the *CsF3′5′H2* promoter as bait to screen for upstream regulators by Y1H. While previous studies identified *CsHSFA1b* and *CsHSFA2* as regulators of *CsJAZ6* expression that modulate catechin accumulation (Zhang et al., 2023), our study identifies *CsHSF24* as a direct transcriptional activator of *CsF3′5′H2*. Targeted Y1H, EMSA, and dual‐luciferase reporter assays confirmed that CsHSF24 directly binds to and activates the *CsF3′5′H2* promoter. Importantly, our EMSA results identified the HSE motif 5’‐GAAAAGTTCT‐3′ (−263 to −254 bp relative to the translation start site) within the *CsF3′5′H2* promoter as the specific binding site of CsHSF24: a DNA–protein complex was formed only with the probe carrying this intact motif (probe 3), whereas the HSE‐mutated probe (probe 4) and the non‐HSE probes failed to bind, providing a mechanistic basis for its regulatory function. The expression pattern of *CsHSF24* across cultivars was consistent with that of *CsF3′5′H2*, suggesting that *CsHSF24* is a key positive regulator of EGCG biosynthesis. Heterologous overexpression of *CsHSF24* in tobacco significantly increased the contents of multiple flavonoids and upregulated downstream flavonoid biosynthetic genes (*NtFLS, NtANS*). These results indicate that CsHSF24 promotes flavonoid biosynthesis across species. Although tobacco lacks the galloyltransferase activity required for EGCG synthesis, the broad upregulation of flavonoid intermediates, particularly dihydromyricetin which is the direct precursor of EGCG biosynthesis, strongly supports the conserved function of *CsHSF24* as a positive regulator of the flavonoid pathway. Further analyses in *CsHSF24*‐silenced tea plants and CsHSF24‐overexpressing calli confirmed that *CsHSF24* positively regulates EGCG biosynthesis by directly modulating *CsF3′5′H2* expression. These findings align with the emerging recognition that HSF transcription factors, such as PhHSF19 in Petunia, can serve as regulators of specialized metabolic pathways beyond their canonical roles in stress responses (Fu et al., 2022). It should be noted that the cultivar‐dependent differences in *CsHSF24* expression were relatively modest compared with those of *CsF3′5′H2* (Figures 1B–D and 2A), suggesting that CsHSF24 contributes to *CsF3′5′H2* expression in an additive rather than a solely determinative manner. In “SQ,” the higher expression of CsRNF (Figure 6G) may further reduce CsHSF24 protein accumulation, and the 14‐bp promoter polymorphism provides an additional, promoter‐intrinsic explanation for the low *CsF3′5′H2* expression in this cultivar.

### CsRNF ubiquitinates CsHSF24 and targets it for degradation via the 26S proteasome pathway

Ubiquitination modification, one of the types of PTMs, can directly affect the activity and location of proteins and regulate a variety of cell activities, including cell cycle, apoptosis, transcriptional regulation, DNA damage repair, and immune response (Sadanandom et al., 2012). In addition, the ubiquitin‐proteasome system (UPS) is a highly efficient protein degradation pathway, which is responsible for the specific degradation of most intracellular proteins. The UPS contains ubiquitin, the ubiquitin‐activating enzyme E1, the ubiquitin‐conjugating enzyme E2, the ubiquitin ligase E3, the 26S proteasome, and deubiquitinating enzymes. The function of ubiquitin ligase E3 is to connect ubiquitin, which is bound to E2, to the target protein to promote the specific recognition and degradation of target protein by 26S proteasome (Sadanandom et al., 2012; Stone, 2014). The ubiquitin‐proteasome pathway plays key roles in plant growth, development, and stress responses (Hao et al., 2021; Lee & Kim, 2011; Yu, Kang, et al., 2021). However, the mechanism of protein post‐translational modification regulating catechin biosynthesis in tea plants is still rarely reported, especially the role of ubiquitin‐proteasome pathway in catechins biosynthesis have not been identified to date. E3 ligases can be largely divided into two groups including single subunit E3 ligases and multi subunit E3 ligases according to their structure. Then, single subunit E3 ligases are further classified into different families based on the presence of specific domains: really interesting new gene (RING) domains, homology with the C‐terminus (HECT) of E6AP, and U‐box (Lee & Kim, 2011). In Arabidopsis, the RING‐type E3 ligases RGLG1 and RGLG2 ubiquitinate *MAPKKK18* to promote its degradation, thereby modulating drought tolerance (Yu, Kang, et al., 2021). Notably, in pear, reduced proteasome‐mediated degradation of the B‐class HSF *HsfB2a* at higher temperatures inhibits anthocyanin synthesis (Wang et al., 2025), demonstrating that ubiquitin‐proteasome control of HSFs directly shapes flavonoid metabolism. In apple, *MdPUB24* ubiquitinates *MdNAC72*, promoting its degradation and consequently upregulating MdPG1 expression to accelerate fruit softening (Wei et al., 2023).

Sequence analysis showed that the E3 ubiquitin ligase we screened belongs to the RING‐finger type, and we named it CsRNF. Pull‐down assays confirmed that CsRNF interacts with CsHSF24 in vitro, and in vitro ubiquitination assays showed that CsRNF ubiquitinates CsHSF24. In *Arabidopsis*, the E3 ligases RGLG1 and RGLG2 could ubiquitinate *MAPKKK18* and promote its degradation; overexpression of *MAPKKK18* in the *rglg1rglg2* double mutant resulted in a significantly lower water loss rate and stronger drought tolerance compared with *MAPKKK18* overexpression in the corresponding WT background (Yu, Kang, et al., 2021). In this study, silencing CsRNF in tea plants resulted in a significant increase in EGCG content, whereas *CsHSF24* transcript levels were not significantly changed in the CsRNF‐silenced plants, consistent with the post‐translational mode of regulation. Our findings establish a functionally analogous regulatory paradigm in tea plants: CsRNF ubiquitinates and promotes the degradation of CsHSF24, thereby reducing *CsF3′5′H2* expression and EGCG biosynthesis. This convergence of regulatory logic across diverse plant species and physiological contexts underscores the evolutionary significance of ubiquitin‐mediated control of transcription factor stability in plant metabolic regulation. Furthermore, higher CsRNF expression in “SQ” compared with high‐EGCG cultivars suggests that enhanced CsHSF24 degradation may be an additional factor contributing to the low‐flavanol phenotype in this cultivar. Consistent with this model, *CsHSF24* overexpression upregulated *CsDFR* and *CsANR* in addition to *CsF3′5′H1* and *CsF3′5′H3*, and increased the contents of all six catechin fractions, with particularly strong increases in ECG, GCG, and GC, whereas *CsHSF24* silencing produced the opposite effects (Figures S6 and S7). The broad increase in catechin contents upon CsHSF24 overexpression indicates that stabilized *CsHSF24* enhances flux through the catechin biosynthetic pathway as a whole. It is noteworthy that HSF proteins are typically activated under heat stress conditions (Hahn et al., 2011; Nover et al., 2001). Whether CsHSF24 protein stability or CsRNF expression is modulated by temperature or other environmental cues, and whether such regulation has adaptive significance for tea plant metabolism, represents an important direction for future investigation.

### A natural 14‐bp deletion in the *CsF3′5′H2* promoter functions as a structural element for transcriptional activation

A key finding of our study is the identification of a natural 14‐bp deletion (ATTACTACACCACC) in the *CsF3′5′H2* promoter that distinguishes the low‐EGCG “SQ” cultivar from high‐EGCG cultivars. Crucially, although this deletion is located approximately 450 bp upstream of the canonical HSE motif and does not disrupt the *CsHSF24* binding site perse, its likely functions as a critical “structural hinge” mediating DNA flexibility. This structural perturbation impairs the precise three‐dimensional chromatin architecture required for effective *CsHSF24*‐mediated transcriptional activation of *CsF3′5′H2*. This discovery establishes a direct mechanistic link between natural genetic variation and metabolic diversity: The promoter polymorphism disrupts a specific spatial regulatory element, thereby abolishing the functional output of a defined transcription factor. This finding bridges the gap between genetic polymorphism and metabolic phenotype, enriching our understanding of the molecular basis governing catechin diversity in tea plants (Shao et al., 2026; Zhang et al., 2020; Zhang et al., 2025). Furthermore, the elucidation of the CsRNF‐CsHSF24‐CsF*3′5′*H2 regulatory axis provides a comprehensive framework integrating transcriptional activation (*CsHSF24*), post‐translational control (*CsRNF*), and natural genetic variation (the 14‐bp structural deletion) in the regulation of EGCG biosynthesis. Interestingly, the 14‐bp sequence (ATTACTACACCACC) is AC‐rich and thus resembles the binding sites of R2R3‐MYB transcription factors implicated in anthocyanin and proanthocyanidin biosynthesis. It is therefore plausible that, in addition to its proposed role as a structural element, this sequence contributes to promoter activity by serving as a target for additional transcriptional activators; Y1H screening using the 14‐bp element as bait may help to identify such factors in the future.

### The CsRNF‐CsHSF24‐CsF3′5′H2 regulatory module orchestrates EGCG biosynthesis through the convergence of transcriptional regulation and post‐translational control

Our study elucidates a multi‐layered regulatory network governing EGCG biosynthesis in tea plants, in which the transcription factor *CsHSF24* directly activates *CsF3′5′H2* expression and the E3 ubiquitin ligase CsRNF negatively controls the stability of the CsHSF24 protein through ubiquitination and proteasomal degradation. This CsRNF–CsHSF24–CsF3′5′H2 module finetunes EGCG accumulation. Superimposed on this regulatory axis, the 14‐bp promoter deletion in the “SQ” cultivar dampens this positive transcriptional input in a promoter‐intrinsic manner, explaining its constitutive low‐flavanol phenotype. Together, these findings bridge a specific genetic polymorphism to a key metabolic trait and enrich our understanding of the molecular basis for catechin diversity in tea plants. Plant regulatory networks are highly complex and coordinated by multiple factors. While manipulation of the core CsRNF‐CsHSF24‐CsF3′5′H2 module consistently altered EGCG levels, its effects on other catechin subtypes were less uniform. This suggests that the biosynthesis of different catechins is likely governed by additional, branch‐specific factors operating independently or in parallel with this pathway. Further research is needed to elucidate the integrated regulatory landscape shaping the complete catechin profile in tea plants.

In conclusion, our study elucidates a multi‐layered regulatory framework governing EGCG biosynthesis in tea plants, centered on the CsRNF‐CsHSF24‐CsF3′5′H2 module. We demonstrate that the transcription factor CsHSF24 directly activates *CsF3′5′H2* expression, thereby promoting EGCG accumulation. Conversely, the E3 ubiquitin ligase CsRNF negatively regulates this pathway by mediating the ubiquitination and degradation of CsHSF24. Importantly, we identify a natural 14‐bp deletion in the *CsF3′5′H2* promoter of the “SQ” cultivar, which attenuates promoter activity and constitutively dampens this positive transcriptional input, providing a molecular explanation for its low‐flavanol phenotype. These findings bridge a specific genetic polymorphism to a key metabolic trait and advance our understanding of catechin diversity in tea plants. The insights gained not only clarify a key regulatory layer in flavonoid metabolism but also offer potential molecular targets for future breeding strategies aimed at tailoring catechin profiles in tea and other plants.

## MATERIALS AND METHODS

### Plant materials and growth condition

One wild tea plant (C. tachangensis, “Siqiu” (“SQ”)) and three cultivars (C. sinensis cv. “Wuniuzao” (WNZ), “Longjing43” (LJ43), and “Zhongcha108” (ZC108)) cultivated in Puan County, Guizhou, China, were used to analyze the accumulation of epigallocatechin gallate (EGCG). C. sinensis cv. “Fudingdabaicha” (FDDB), grown at Guizhou University in a natural field, was also used as experimental material. “Fudingdabaicha” (FDDB) plants used in this work were grown in a growth room (25°C/18°C, day/night) with a relative humidity of 70%. Buds, old leaves, fruits, flowers, stems, and roots from FDDB were collected, immediately frozen in liquid nitrogen, and stored at −80°C until further analysis. FDDB was also used as the source material for cloning the coding sequences of CsHSF24 and CsRNF and the promoter fragments used in the Y1H library screen, for the VIGS experiments, and for generating transgenic tea calli and tobacco plants. The four cultivars from Puan County were used for the comparative metabolite and expression analyses, and their respective genomic DNAs were used for cloning and comparing the CsF3′5′H1 and CsF3′5′H2 coding and promoter regions. The use of different genetic backgrounds reflects the fact that the callus and VIGS systems were established in FDDB, whereas the cultivar comparison required field‐grown plants of “SQ,” “WNZ,” “LJ43,” and “ZC108.” Sequence analysis and promoter alignment (Figure S13) showed that the CsF*3′5′*H2 promoter of “Fudingdabaicha” contains the 14‐bp segment (ATTACTACACCACC) and thus resembles the alleles of the high‐EGCG cultivars “WNZ,” “LJ43,” and “ZC108” rather than the “SQ” allele. In addition, alignment of the CsF*3′5′*H2 coding sequences (1533 bp) showed that they are identical among the five cultivars (Figure S14), consistent with Figure S5.

### Measurement of catechins contents

The samples were subjected to blanching treatment, then dried to constant weight and grated. Extract 10 mg of freeze‐dried samples with 5 mL methanol (70%), and the supernatant was transferred to the volumetric bottle after extraction. The catechins extracts were detected using a Thermo UltiMate UHPLC system as in the previously described method (Su et al., 2020). All data are quantified according to their respective standard curves. The specific catechins (EGCG) content was expressed as mg/g of dry weight (mg/g DW).

### Total RNA and gDNA extraction, cDNA synthesis, and quantitative real‐time PCR (RT‐qPCR) analysis

Total RNA and gDNA were extracted from samples using the Trizol reagent (TaKaRa, Dalian, China) and a DNAsecure Plant Kit (Tiangen, Beijing, China), and the former then converted into cDNAs using Prime Script RT reagent kit (Vazyme, Nanjing, China) according to the manufacturer's instructions. RT‐qPCR was performed using the SYBR qPCR Master Mix (Vazyme, Nanjing, China) according to the following program: 95°C for 30 s, followed by 40 cycles at 95°C for 10 s; 6°C for 30 s, and *CsActin* (XM_028240327.1) was used as the internal control. Three biological replicates were performed for each experiment. All primers used in this study, including those for RT‐qPCR (*CsF3′5′H1, CsF3′5′H2, CsF3′5′H3, CsDFR, CsLAR, CsANS, CsANR, CsHSF24, CsRNF*, and Actin), vector construction (including VIGS inserts in pTRV2 with EcoRI/XhoI ends and BiFC constructs), subcellular localization, EMSA probe preparation (probes 1–4, HSE‐mutant probes, and unlabeled competitors), and pull‐down assays, are listed in Table S1.

### Sequence alignment and phylogenetic analyses

Conserved domains were analyzed using the Conserved Domain Search Service (CD Search, https://www.ncbi.nlm.nih.gov/Structure/cdd/wrpsb.cgi). Deduced amino acid sequences of HSF or RNF proteins from other plant species were obtained from the NCBI database (https://www.ncbi.nlm.nih.gov). Sequence alignment was performed using DNAMAN 6.0 software.

### Subcellular localization of CsHSF24

The full‐length open reading frame (ORF) of CsHSF24 without the stop codon was PCR‐amplified using gene‐specific primers (Table S1) and inserted into the GFP‐fusion expression vector pCAMBIA1300 to generate the 35S:CsHSF24‐GFP construct. The fusion construct was transiently expressed in tobacco (*Nicotiana benthamiana*) leaf epidermal cells via Agrobacterium tumefaciens‐mediated transformation. At 24 h post‐infiltration, fluorescent signals were visualized using a confocal laser scanning microscope (Leica TCS SP8, Germany). Primers used are listed in Table S1.

### Yeast one‐hybrid (Y1H) assay

To screen for transcription factors interacting with the CsF3′5′H2 promoter, a Y1H screen was performed using the Aureobasidin A (AbA) system. The promoter sequence of *CsF3′5′H2* (2000 bp) was cloned into the pAbAi vector (Clontech) to generate the bait construct, pCsF*3′5′*H2‐Pro‐AbAi. This bait plasmid was linearized and integrated into the genome of the Y1HGold yeast strain to create the reporter strain, Y1HGold [pCsF3′5′H2‐Pro‐AbAi]. The minimum inhibitory concentration of AbA for this reporter strain was determined on SD/‐Ura plates supplemented with increasing concentrations of AbA (up to 500 ng/mL). For the library screening, a cDNA library was constructed from tea plant leaf mRNA in the pGADT7‐Rec2 vector (Clontech). This prey library was then transformed into the generated reporter strain. Positive clones were selected on synthetically defined (SD) medium lacking tryptophan (−Trp) and supplemented with a stringent concentration of 500 ng/mL AbA. To confirm the specific interaction between the *CsF3′5′H2* promoter and the transcription factor *CsHSF24*, the coding sequence (CDS) of CsHSF24 was cloned into the pGADT7‐AD vector to generate the prey construct, pGADT7‐AD‐CsHSF24. This construct and the empty pGADT7‐AD vector (negative control) were separately transformed into the reporter strain. The interactions were assessed by spotting serial dilutions of transformed yeast cells on SD/−Trp medium (for growth control) and on SD/−Trp medium containing a selective concentration of AbA. Bait constructs for the *CsF3′5′H1* and *CsF3′5′H3* promoters were generated in the same way and screened against the same cDNA library; the inserts of the positive clones were sequenced and are listed in Tables S2 and S3.

### Dual‐luciferase reporter assay

The full‐length coding sequence (CDS) of *CsHSF24* was amplified and cloned into the pGreenII 62‐SK vector under the control of the CaMV 35S promoter to generate the effector construct pGreenII‐62‐SK‐35S‐*CsHSF24*. The promoter fragments (approximately 2000 bp upstream of the translation start site) of *CsF3′5′H1* and *CsF3′5′H2* were amplified from genomic DNA and individually inserted into the pGreenII 0800‐LUC vector to generate the reporter constructs pGreenII 0800‐RLuc‐*CsF3′5′H1*‐Pro::FLuc and pGreenII 0800‐RLuc‐*CsF3′5′H2*‐Pro::FLuc, in which the *CsF3′5′H1* or *CsF3′5′H2* promoter drives the expression of firefly luciferase (FLuc). The Renilla luciferase (RLuc) gene driven by the CaMV 35S promoter within the same pGreenII 0800‐LUC vector served as an internal control for normalization of transfection efficiency. The effector and reporter constructs were individually transformed into Agrobacterium tumefaciens strain GV3101 (pSoup). Positive transformants were cultured, harvested, and resuspended in infiltration buffer (MS medium supplemented with 10 mM MES, pH 5.6, 10 mM MgCl_2_, and 200 μM acetosyringone) to an OD_600_ of 0.6–0.8. The agrobacterial suspensions harboring the effector and reporter constructs were mixed in appropriate ratios and infiltrated into the abaxial side of 4‐week‐old *Nicotiana benthamiana* leaves using a needleless syringe. After infiltration, plants were incubated in darkness for 3 days. For luciferase imaging, the infiltrated leaves were sprayed with 0.2 mg/mL D‐fluorescein (containing 0.01% Triton X‐100) and incubated in the dark for 20–30 min to quench chlorophyll fluorescence. Luciferase signals were captured using a chemiluminescence imaging system (Fusion FX7, VILBER). For quantitative analysis, leaf discs were collected from the infiltrated regions, and luciferase activities were measured using the Dual‐Luciferase Reporter Assay System (Promega, Cat. No. PR‐E1910) according to the manufacturer's instructions. The firefly luciferase (LUC) activity was normalized to the Renilla luciferase (REN) activity, and the relative LUC/REN ratio was calculated. At least six biological replicates were performed for each treatment combination (Zhang et al., 2021).

### Electrophoretic mobility shift assay (EMSA)

The ORF of *CsHSF24* was PCR‐amplified and subcloned into the pGEX‐4T‐1 vector to express GST‐tagged CsHSF24 (a His‐tagged version bound probes non‐specifically in preliminary assays and was therefore replaced); recombinant protein expression and EMSA were then conducted according to the method previously described (Yao et al., 2023). Primers used are listed in Table S1. Five unlabeled (cold) DNA probes spanning the CsF*3′5′*H2 promoter region were synthesized (probe 1: −1641 to −1608 bp; probe 2: −1699 to −1666 bp; probes 3: −270 to −239 bp; probe 4: −418 to −387 bp relative to the start codon ATG). Probe 3 contains the HSE motif 5′‐GAAAAGTTCT‐3′ (−263 to −254 bp), and probe 5 is identical to probe 3 except for six base substitutions in the HSE core. EMSAs were performed using purified GST‐CsHSF24 protein and an unlabeled DNA fragment from the *CsF3′5′H2* promoter. Equal amounts of the wild‐type probe ((100×) fmol per reaction) were incubated with increasing amounts of GST‐CsHSF24 (0.05 (5×), 0.1 (10×), 0.5 (50×), 1 (100×), or 2 μg (200×)) in 1× binding buffer (composition, e.g., 10 mM Tris‐HCl, 50 mM KCl, 1 mM DTT, 5% glycerol, pH 7.5) containing (poly(dI‐dC), 1 μg) as nonspecific competitor at (25 °C for 20 min). Purified GST protein (1 μg, 100×) was incubated with the probe as a negative protein control. To test sequence specificity, a mutant probe carrying six base substitutions in the HSE core was incubated with GST‐CsHSF24 (1 μg, 100×). The reaction mixtures were resolved on a native (5%) polyacrylamide gel in (0.5× TBE), and DNA was visualized by GelRed staining. All probe sequences are listed in Supplementary Table S1.

### Bimolecular Fluorescence Complementation (BiFC) Assay

The full‐length coding sequences (CDS) of “*CsHSF24*” and *“CsRNF*” were amplified and cloned into the pCAMBIA1300‐35S‐Myc‐YFPN (N‐terminal fragment of yellow fluorescent protein) and pCAMBIA1300‐35S‐HA‐YFPC (C‐terminal fragment of YFP) vectors, respectively (Waadt et al., 2008). The resulting constructs were designated YFPN‐CsHSF24 and YFPC‐CsRNF; the full‐length CDSs were amplified with the *CsHSF24* and *CsRNF* genome‐amplification primers listed in Table S1.

Agrobacterium tumefaciens strain GV3101 carrying the appropriate combinations of BiFC constructs (YFPN‐*CsHSF24* + YFPC‐*CsRNF*, YFPN‐CsHSF24 + YFPC‐EV, YFPN‐EV + YFPC‐*CsRNF*, or YFPN‐EV + YFPC‐EV as negative controls) were resuspended in infiltration buffer (MS + 10 mM MES, pH 5.6, 10 mM MgCl_2_, 200 μM acetosyringone) to an OD_600_ of 0.6–0.8. The bacterial suspensions were mixed in equal volumes and infiltrated into the abaxial side of 4‐week‐old *Nicotiana benthamiana* leaves using a needleless syringe. After 48–72 h of incubation, YFP fluorescence was visualized using a confocal laser scanning microscope (LSM 880; Carl Zeiss, Jena, Germany). YFP was excited at 514 nm, and emission was detected between 525 and 550 nm.

### Yeast two‐hybrid (Y2H) library screening

To identify proteins that interact with CsHSF24, a Y2H screen was performed using the MATCHMAKER Gold Yeast Two‐Hybrid System (Clontech). The full‐length CDS of CsHSF24 was cloned into pGBKT7 to generate the bait plasmid pGBKT7‐CsHSF24, and the bait was verified to have no auto‐activation or toxicity in yeast strain Y2HGold. A cDNA library was constructed from tea plant (FDDB) leaf mRNA in the pGADT7 prey vector (Clontech). The prey library was introduced into Y2HGold cells harboring pGBKT7‐CsHSF24 by the lithium acetate method, and transformants were plated on SD/‐Leu/‐Trp medium. Positive clones were selected on SD/‐Ade/‐His/‐Leu/‐Trp medium supplemented with X‐α‐Gal, and the prey inserts of the positive clones were PCR‐amplified and sequenced. A total of 34 positive clones were obtained, all of which encoded the same RING‐finger E3 ubiquitin ligase, which we named CsRNF (Table S4). The interaction between full‐length CsHSF24 and CsRNF was subsequently confirmed by directed Y2H, BiFC, and pull‐down assays.

### Yeast Two‐Hybrid Assay

The full‐length CDS of CsRNF was cloned into the pGBKT7 vector (DNA‐binding domain vector), and the full‐length CDS of CsHSF24 was cloned into the pGADT7 vector (activation domain vector). The resulting plasmids (pGBKT7‐CsRNF and pGADT7‐CsHSF24) were co‐transformed into yeast strain AH109 using the lithium acetate method. Transformants were first selected on synthetic dropout (SD) medium lacking leucine and tryptophan (SD/‐Leu/‐Trp). Positive colonies were then transferred to SD medium lacking adenine, histidine, leucine, and tryptophan (SD/‐Ade/‐His/‐Leu/‐Trp) to assess protein–protein interactions (Li, Li, et al., 2022). The combinations pGADT7‐RecT + pGBKT7‐53 and pGADT7‐RecT + pGBKT7‐Lam were used as positive and negative controls, respectively. To evaluate the strength of interactions, serial dilutions (10^−1^, 10^−2^, 10^−3^, 10^−4^, 10^−5^) of yeast cultures were spotted onto the selective media.

### Validation of protein–DNA interactions via agrobacterium‐mediated transient expression and Dual‐Luciferase Assay

The promoter region of *CsF3′5′H2* (approximately 2 kb upstream of the translation start site) was amplified from genomic DNA and cloned into the pGreenII 0800‐RLuc‐MCS vector, driving the expression of firefly luciferase (FLuc) as the reporter gene. The Renilla luciferase (RLuc) gene driven by the CaMV 35S promoter in the same vector was used as an internal control for normalization. The full‐length CDS of CsRNF and CsHSF24 was individually cloned into the pGreenII‐62‐SK vector under the control of the CaMV 35S promoter to generate effector constructs 35S‐CsRNF (Effector 1) and 35S‐CsHSF24 (Effector 2). The *A. tumefaciens* (GV3101, pSoup) strain carrying the reporter protein and effector construct was cultured to OD_600_ = 0.6–0.8 and resuspended in the infiltration buffer. Then mix according to the following combination:

$$\text{V1}:\text{Reporter}+\text{Effector}\;1+\text{Empty Vector}\;\left(\mathrm{EV}\right)$$

V2:Reporter+Effector2+EV

V3:Reporter+Effector1+Effector2

$$\begin{array}{l} \text{V4}:\text{Negative Control}(\mathrm{NC},\text{pGreenII}\;0800‐\text{RLuc}‐\mathrm{MCS}\\ \;\text{without promoter})+\mathrm{EV} \end{array}$$

The OD_600_ ratio of reporter to effector was 1:1:1. The mixed bacterial suspensions were infiltrated into *N. benthamiana* leaves as described above (Zhang et al., 2021). After 48 h, leaf discs were collected, and luciferase activities were measured using the Dual‐Luciferase Reporter Assay System (Promega, Madison, WI, USA) according to the manufacturer's instructions. The FLuc activity was normalized to the RLuc activity, and the relative luciferase activity (FLuc/RLuc) was calculated. At least six biological replicates were performed for each treatment.

### In vivo ubiquitination of CsHSF24 by CsRNF was validated by a dual‐luciferase assay with MG132 treatment

To investigate whether CsRNF regulates CsHSF24 via the 26S proteasome pathway, a proteasome inhibitor MG132 was applied in the dual‐luciferase assay (Yan et al., 2024). *A. tumefaciens* infiltration was performed as described above with the following combinations:

V5:Reporter+Effector1+Effector2+Water

V6:Reporter+Effector1+Effector2+MG132

V7:Reporter+Effector1+Effector2+DMSO

V8:Reporter+Effector2+EV+MG132

After 24 h of Agrobacterium infection, MG132 (Sigma‐Aldrich, St.Louis, MO, USA) dissolved in dimethyl sulfoxide (DMSO) was prepared into a solution of 50 μM and immersed in the same leaves. Water and DMSO were used as an additional control. After another 24 h of incubation, luciferase activities were measured as described above.

### Pull‐down assay

The CDS of *CsHSF24* and *CsRNF* was recombined into the vector pGEX‐4 T‐1 (GST tag) and pET28 (His tag), respectively. Next, pGEX‐4 T‐1‐*CsHSF24* and pET28‐*CsRNF* were transformed together into BL21(DE3) to induce protein expression. Then, 20 μL pre‐washed GST magnetic beads were added to the protein‐containing supernatant and incubated at 4°C for 4 h. After incubation, the GST magnetic beads were eluted, and the interaction between CsHSF24 and CsRNF was detected by Western Blot. Primers used are listed in Table S1.

### In *vitro* ubiquitination assay

E3 Ligase Auto‐Ubiquitylation Assay Kit (Abcam, UK) was used to detect the ubiquitination in *vitro*. Briefly, the pGEX‐4 T‐1‐*CsHSF24* and pET28‐*CsRNF* were transformed into *Escherichia coli BL21* (*DE3*) to induce protein expression and purification, respectively. Then, reaction components were added according to the manufacturer's instructions, including ATP, ubiquitin (Ub), Ub‐activating enzyme (E1), Ub‐conjugating enzyme (E2), 100 ng of E3 (purified CsRNF), and 300 ng of purified CsHSF24 protein, and ubiquitination reaction buffer, which were mixed and incubated at 37°C for 1 h. The proteins were separated by SDS‐PAGE, and ubiquitinated CsHSF24 protein was detected using an anti‐ubiquitin antibody.

### Construction and transformation of tea callus and tobacco

To silence *CsF3′5′H2*, *CsHSF24*, or *CsRNF* expression in tea plants, 300‐bp partial CDS fragments were cloned into the pTRV2 vector using EcoRI/XhoI to generate constructs (pTRV2‐*CsF3′5′H2*, pTRV2‐*CsHSF24*, pTRV2‐*CsRNF*); the cloning primers are listed in Table S1. Each recombinant plasmid was transformed into *Agrobacterium* strain using the heat shock method. The pTRV2 empty vector was used as the negative control. VIGS was performed in tea plantlets of “Fudingdabaicha” according to a previously described method (Li et al., 2023). In addition, the *CsHSF24* CDS was inserted into the pSH737‐35S vector to generate the overexpression construct pSH737‐35S‐*CsHSF24*, which was transformed into A. tumefaciens strain GV3101. Transgenic tea calli and tobacco (Nicotiana tabacum cv. Xanthi) overexpressing CsHSF24 were generated by A. tumefaciens‐mediated transformation. In brief, the leaves of “Fudingdabaicha” were used as explant, after being treated with *A. tumefaciens* infection solution (OD_600_ = 0.6 with 100 μmoL·L^−1^ acetosyringone (AS)) for 10 min; they were co‐cultured in MS medium with 0.5 mg·L^−1^ 6‐benzylaminopurine (6‐BA) and 1.0 mg·L^−1^ 1‐naphthaleneacetic acid (NAA) in dark. After 3 days of co‐culture, they were transferred to screening medium (MS + 0.5 mg·L^−1^ 6‐BA + 1.0 mg·L^−1^ NAA + 200 mg·L^−1^ timentin (Tim) + 30 mg·L^−1^ kanamycin). After about 60 days, kanamycin‐resistant overexpression calli were obtained and further confirmed by RT‐PCR. The expression level of *CsHSF24* in the positive transgenic lines was examined by RT‐qPCR. Primers used are listed in Table S1.

### Statistical analysis

All experiments were performed with at least three biological replicates. Data were statistically analyzed using SPSS 20.0 software (SPSS Inc., Chicago, IL, USA). Statistical differences in gene expression and physiological parameters between different genotypes were assessed using Tukey's multiple range test (*P* < 0.05).

## Accession numbers

*CsF3′5′H1* (LOC114261298), *CsHSF24* (LOC114312113), *CsRNF* (LOC114275218), *AcHSF* (PSR87978.1), *NtHSF24* (XP_009589845.1), *PtHSF* (XM_006377285.3), *VrHSF24* (XP_034691502.1), *ZjHSF24* (XP_015890148.2), *NsRNF* (KAA8528524.1), *AcRNF* (PSS31436.1), *GhRNF* (XP_016749299.1), *ItRNF* (XP_031117831.1).

## Author Contributions

XY: Conceptualization, visualization, supervision, resources, project administration. HC: Conceptualization, formal analysis, writing—review and editing, supervision. HA: Formal analysis, data curation, visualization. YH: Investigation, writing—original draft. TL: Visualization, writing—review and editing. QZ: Investigation, formal analysis. CL: writing—review and editing, supervision. LL: Resources, visualization, investigation, writing—review and editing. All authors have read and approved the final version of the manuscript.

## Conflict of Interest

The authors declare that they have no known competing financial interests or personal relationships that could have appeared to influence the work reported in this paper.

## Supporting information


**Figure S1.** The expression level of *CsF3'5'H1 CsF3'5'H2 and CsF3'5'H3* in different tissues (bud, leaf, steam, root, flower, and pericarp) from “WNZ.”
**Figure S2.** Multiple sequence alignment of CsHSF24 and HSFs from other plants species. The proteins are from *Actinidia chinensis* (*AcHSF*, PSR87978.1), *Nicotiana tomentosiformis* (*NtHSF24*, XP009589845.1), *Populus trichocarpa* (*PtHSF*, XM006377285.3), *Vitis riparia* (*VrHSF*, XP034691502.1), and *Ziziphus jujuba* (*ZjHSF*, XP015890148.2). The conserved DNA‐binding domain is indicated by red box.
**Figure S3.** Preliminary EMSA with His‐tagged CsHSF24 and expression/purification of the recombinant HSF‐GST fusion protein. EMSA (Left) examining the binding of His‐tagged CsHSF24 to the CsF3*'*5*'*H2 promoter probe. The probe was incubated alone (lane 1), with the His tag alone (lane 2) or with His‐tagged CsHSF24 (lanes 3–7), and the reaction mixtures were separated on a native polyacrylamide gel. Shifted bands indicate probe–protein complexes. Because a shifted band was also observed when the probe was incubated with the His tag alone (lane 2), indicating non‐specific binding between the probe and the His tag, a GST fusion system was adopted for the subsequent EMSA experiments shown in Figure 2f. SDS‐PAGE *(Right)* analysis of recombinant GST and HSF‐GST fusion proteins expressed in *E. coli*. The HSF‐GST fusion protein (~58 kDa) was detected in the soluble fraction, wash, and eluate fractions, with GST alone (~26 kDa) as a control, confirming successful expression and purification of the fusion protein used for the subsequent EMSA.
**Figure S4.** TRV‐mediated VIGS system was used to silence the CsHSF24 expression in tea plants. The phenotypes of WT, pTRV, and CsHSF24‐sliencing (pTRV2‐CsHSF24‐1, pTRV2‐CsHSF24‐2, and pTRV2‐CsHSF24‐3) plants under normal condition.
**Figure S5.** Overexpression of *CsHSF24* promotes tea callus growth and enhances GUS activity.
**Figure S6.** *CsHSF24* silencing alters catechin‐pathway gene expression and catechin contents in tea plants. (a–f) Relative expression of *CsDFR*, *CsLAR*, *CsANS*, *CsANR*, *CsF3'5'H1*, and *CsF3'5'H3* in WT, pTRV2, and *CsHSF24‐*silenced (pTRV2‐*CsHSF24*) tea plants; (g–l) contents of GC, EGC, C, EC, GCG, and ECG in WT, pTRV2, and *CsHSF24‐*silenced tea plants. Error bars: ±SD. Statistical significance was determined using Tukey's multiple range test (*P* < 0.05), with different lowercase letters indicating significant differences.
**Figure S7.** *CsHSF24* overexpression catechin‐pathway gene expression and catechin contents in tea plants. (a–f) Relative expression of *CsDFR*, *CsLAR*, *CsANS*, *CsANR*, *CsF3'5'H1*, and *CsF3'5'H3* in control and CsHSF24‐overexpressing (CsHSF24‐OE) tea calli; (m–r) contents of GC, EGC, C, EC, GCG, and ECG in WT, pTRV2, and CsHSF24‐silenced tea plants; (s–x) contents of GC, EGC, C, EC, GCG, and ECG in control and CsHSF24‐OE calli. Error bars: ±SD (*n* = 3). Statistical significance was determined using Tukey's multiple range test (*P* < 0.05), with different lowercase letters indicating significant differences.
**Figure S8.** The coding sequence of *CsF3'5'H2* is conserved, whereas its promoter is polymorphic among cultivars. (a) Alignment of the *CsF3'5'H2* gene coding region (CDS) sequences from the five tea plant cultivars. (b) Alignment of the *CsF3'5'H2* gene promoter sequences (approximately 2.0 kb upstream of the transcription start site) from the five tea plant cultivars.
**Figure S9.** Alignment of the *CsF3'5'H1* gene promoter sequences (approximately 2.0 kb upstream of the transcription start site) from the five tea plant cultivars.
**Figure S10.** The effect of *CsHSF24* overexpressing in tobacco flavonoids accumulation. (A) The phenotypes of WT and transgenic tobacco overexpressing *CsHSF24* (TP‐1, TP‐2, TP‐3) under normal condition. (B–F) The content of rutin (B), quercetin (C), myricetin (D), dihydromyricetin (E) and dihydroquercetin (F) in WT and transgenic tobacco (TP‐1, TP‐2, TP‐3). Error bars: ±SD. Statistical significance was determined using Tukey's multiple range test (*P* < 0.05).
**Figure S11.** The expression of flavonoids biosynthesis‐related genes in effect of *CsHSF24* overexpressing tobacco. (A–D) The expression of *NtANS* (A), *NtFLS* (B), *NtF3H* (C), and *NtCHI* (D) in WT and transgenic tobacco (TP‐1, TP‐2, TP‐3). Error bars: ±SD. Statistical significance was determined using Tukey's multiple range test (*P* < 0.05).
**Figure S12.** The expression of flavonoids biosynthesis‐related genes in effect of *CsHSF24* overexpressing tobacco. The expression of *NtDFR, NtLAR, NtANR, NtFLS, NtF3H, and NtCHI* in WT and transgenic tobacco (TP‐1, TP‐2, TP‐3). Error bars: ±SD. Statistical significance was determined using Tukey's multiple range test (*P* < 0.05).
**Figure S13.** Positive clones of identified. Numbers 1–34 are the 34 initial positive clones singled out, with the number “+” for the positive control and the number “‐” for the negative control.
**Figure S14.** Multiple sequence alignment of CsRNF and RNFs from other plants species. The proteins are from *Nyssa sinensis* (NsRNF, KAA8528524.1), *Actinidia chinensis* (AcRNF, PSS31436.1), *Gossypium hirsutum* (GhRNF, XP_016749299.1), *Ipomoea triloba* (ItRNF, XP_031117831.1), and *Citrus sinensis* (CsRNF, XP_006473305.2).

**Table S1.** Name and sequence of primers and probes used in this study.
**Table S2.** Yeast one‐hybrid screening results using the promoters of CsF3*'*5*'*H1 genes.
**Table S3.** Yeast one‐hybrid screening results using the promoters of CsF3*'*5*'*H2 genes.
**Table S4.** Positive clone sequencing results.

## Data Availability

The authors declare that the data supporting the findings of this study are available within the paper and its supplementary information files.
