# Supplementary material for: A novel CsRNF‐CsHSF24‐CsF3′5′H2 module regulates epigallocatechin gallate biosynthesis in Camellia sinensis
Source: Plant J. 2026 Oct 9;128(1):e71155. doi: 10.1111/tpj.71155 (PMC13651704; doi:10.1111/tpj.71155)
Supplement: Supplementary file 1 [file TPJ-128-0-s002.docx]

**A novel CsRNF-CsHSF24-*CsF3’5’H2* module regulates epigallocatechin gallate biosynthesis in *Camellia sinensis***

Xinzhuan Yao ^a,b^, Hufang Chen ^a^, Haili An ^a^, Yilan Hu ^a^, Tong Li ^b^, Qi Zhao ^a^, Tengxian He^a^, Chao Luo^a^, Xiaofang Zeng^a^, Litang Lu ^a, *^

^a^ College of Life Sciences, Guizhou University, Guiyang 550025, China

^b^ College of Tea Sciences, Institute of Plant Health & Medicine, Guizhou University, Guiyang 550025, China

*Correspondence: ltlv@gzu.edu.cn; Tel.: +86-13639031105

*Corresponding author at: College of Life Science, Guizhou University, Guiyang 550025, People’s Republic of China.

**Figure S1 The expression level of *CsF3’5’H1*** ***CsF3’5’H2 and CsF3’5’H3* in different tissues (bud, leaf, steam, root, flower, and pericarp) from ‘WNZ’.**

**Figure S2 Multiple sequence alignment of CsHSF24 and HSFs from other plants species**

The proteins are from *Actinidia chinensis* (*AcHSF*, PSR87978.1), *Nicotiana tomentosiformis* (*NtHSF24*, XP009589845.1), *Populus trichocarpa* (*PtHSF*, XM006377285.3), *Vitis riparia* (*VrHSF*, XP034691502.1), and *Ziziphus jujuba* (*ZjHSF*, XP015890148.2). The conserved DNA binding domain is indicated by red box.

**Figure S3 Preliminary EMSA with His-tagged CsHSF24 and expression/purification of the recombinant HSF-GST fusion protein.**

EMSA (Left) examining the binding of His-tagged CsHSF24 to the CsF3'5'H2 promoter probe. The probe was incubated alone (lane 1), with the His tag alone (lane 2), or with His-tagged CsHSF24 (lanes 3–7), and the reaction mixtures were separated on a native polyacrylamide gel. Shifted bands indicate probe–protein complexes. Because a shifted band was also observed when the probe was incubated with the His tag alone (lane 2), indicating non-specific binding between the probe and the His tag, a GST-fusion system was adopted for the subsequent EMSA experiments shown in Figure 2f.  SDS-PAGE *(Right)* analysis of recombinant GST and HSF-GST fusion proteins expressed in *E. coli*. The HSF-GST fusion protein (~58 kDa) was detected in the soluble fraction, wash, and eluate fractions, with GST alone (~26 kDa) as a control, confirming successful expression and purification of the fusion protein used for the subsequent EMSA.

**Figure S4 TRV-mediated VIGS system was used to silence the CsHSF24 expression in tea plants.**

The phenotypes of WT, pTRV and CsHSF24-sliencing (pTRV2-CsHSF24-1, pTRV2-CsHSF24-2, and pTRV2-CsHSF24-3) plants under normal condition.

**Figure S5 Overexpression of *CsHSF24* promotes tea callus growth and enhances GUS activity.**

**Figure S6 *CsHSF24* silencing alters catechin-pathway gene expression and catechin contents in tea plants.**

(a-f) Relative expression of *CsDFR*, *CsLAR*, *CsANS*, *CsANR*, *CsF3'5'H1*, and *CsF3'5'H3* in WT, pTRV2, and *CsHSF24-*silenced (pTRV2-*CsHSF24*) tea plants; (g-l) contents of GC, EGC, C, EC, GCG, and ECG in WT, pTRV2, and *CsHSF24-*silenced tea plants. Error bars: ±SD. Statistical significance was determined using Tukey's multiple range test (P < 0.05), with different lowercase letters indicating significant differences.

**Figure S7 *CsHSF24* overexpression catechin-pathway gene expression and catechin contents in tea plants.**

(a-f) Relative expression of *CsDFR*, *CsLAR*, *CsANS*, *CsANR*, *CsF3'5'H1*, and *CsF3'5'H3* in control and CsHSF24-overexpressing (CsHSF24-OE) tea calli; (m-r) contents of GC, EGC, C, EC, GCG, and ECG in WT, pTRV2, and CsHSF24-silenced tea plants; (s-x) contents of GC, EGC, C, EC, GCG, and ECG in control and CsHSF24-OE calli. Error bars: ±SD (n=3). Statistical significance was determined using Tukey's multiple range test (P < 0.05), with different lowercase letters indicating significant differences.

a

b

**Figure S8 The coding sequence of *CsF3’5’H2* is conserved whereas its promoter is polymorphic among cultivars.**

(a) Alignment of the *CsF3’5’H2* gene coding region (CDS) sequences from the five tea plant cultivars. (b) Alignment of the *CsF3’5’H2* gene promoter sequences (approximately 2.0 kb upstream of the transcription start site) from the five tea plant cultivars.

**Figure S9 Alignment of the *CsF3’5’H1* gene promoter sequences (approximately 2.0 kb upstream of the transcription start site) from the five tea plant cultivars.**

**Figure S10 The effect of *CsHSF24* overexpressing in tobacco flavonoids accumulation**

**(A)** The phenotypes of WT and transgenic tobacco overexpressing *CsHSF24* (TP-1, TP-2, TP-3) under normal condition. (B-F) The content of rutin (B), quercetin (C), myricetin (D), dihydromyricetin (E), and dihydroquercetin (F) in WT and transgenic tobacco (TP-1, TP-2, TP-3). Error bars: ±SD. Statistical significance was determined using Tukey’s multiple range test (*P* < 0.05).

**Figure S11 The expression of flavonoids biosynthesis related genes in effect of *CsHSF24* overexpressing tobacco**

(A-D) The expression of *NtANS* (A), *NtFLS* (B), *NtF3H* (C), and *NtCHI* (D) in WT and transgenic tobacco (TP-1, TP-2, TP-3). Error bars: ±SD. Statistical significance was determined using Tukey’s multiple range test (*P* < 0.05).

**Figure S12 The expression of flavonoids biosynthesis related genes in effect of *CsHSF24* overexpressing tobacco**

The expression of *NtDFR, NtLAR, NtANR, NtFLS, NtF3H, and NtCHI* in WT and transgenic tobacco (TP-1, TP-2, TP-3). Error bars: ±SD. Statistical significance was determined using Tukey’s multiple range test (*P* < 0.05).

**Figure S13 Positive clones of identified**

Numbers 1-34 are the 34 initial positive clones singled out, with the number ‘+’ for the positive control and the number ‘-’ for the negative control.

**Figure S14 Multiple sequence alignment of CsRNF and RNFs from other plants species**

The proteins are from *Nyssa sinensis* (NsRNF, KAA8528524.1), *Actinidia chinensis* (AcRNF, PSS31436.1), *Gossypium hirsutum* (GhRNF, XP_016749299.1), *Ipomoea triloba* (ItRNF, XP_031117831.1), and *Citrus sinensis* (CsRNF, XP_006473305.2).
